# Machine learning-based classification of the movements of children with profound or severe intellectual or multiple disabilities using environment data features

**DOI:** 10.1371/journal.pone.0269472

**Published:** 2022-06-30

**Authors:** Von Ralph Dane Marquez Herbuela, Tomonori Karita, Yoshiya Furukawa, Yoshinori Wada, Akihiro Toya, Shuichiro Senba, Eiko Onishi, Tatsuo Saeki

**Affiliations:** 1 Faculty of Education, Center of Inclusive Education, Ehime University, Ehime, Japan; 2 Graduate School of Humanities and Social Sciences, Hiroshima University, Hiroshima, Japan; 3 DigitalPia Co., Ltd., Ehime, Japan; Sreenidhi Institute of Science and Technology, INDIA

## Abstract

Communication interventions have broadened from dialogical meaning-making, assessment approaches, to remote-controlled interactive objects. Yet, interpretation of the mostly pre-or protosymbolic, distinctive, and idiosyncratic movements of children with intellectual disabilities (IDs) or profound intellectual and multiple disabilities (PIMD) using computer-based assistive technology (AT), machine learning (ML), and environment data (ED: location, weather indices and time) remain insufficiently unexplored. We introduce a novel behavior inference computer-based communication-aid AT system structured on machine learning (ML) framework to interpret the movements of children with PIMD/IDs using ED. To establish a stable system, our study aimed to train, cross-validate (10-fold), test and compare the classification accuracy performance of ML classifiers (eXtreme gradient boosting [XGB], support vector machine [SVM], random forest [RF], and neural network [NN]) on classifying the 676 movements to 2, 3, or 7 behavior outcome classes using our proposed dataset recalibration (adding ED to movement datasets) with or without Boruta feature selection (53 child characteristics and movements, and ED-related features). Natural-child-caregiver-dyadic interactions observed in 105 single-dyad video-recorded (30-hour) sessions targeted caregiver-interpreted facial, body, and limb movements of 20 8-to 16-year-old children with PIMD/IDs and simultaneously app-and-sensor-collected ED. Classification accuracy variances and the influences of and the interaction among recalibrated dataset, feature selection, classifiers, and classes on the pooled classification accuracy rates were evaluated using three-way ANOVA. Results revealed that Boruta and NN-trained dataset in class 2 and the non-Boruta SVM-trained dataset in class 3 had >76% accuracy rates. Statistically significant effects indicating high classification rates (>60%) were found among movement datasets: with ED, non-Boruta, class 3, SVM, RF, and NN. Similar trends (>69%) were found in class 2, NN, Boruta-trained movement dataset with ED, and SVM and RF, and non-Boruta-trained movement dataset with ED in class 3. These results support our hypotheses that adding environment data to movement datasets, selecting important features using Boruta, using NN, SVM and RF classifiers, and classifying movements to 2 and 3 behavior outcomes can provide >73.3% accuracy rates, a promising performance for a stable ML-based behavior inference communication-aid AT system for children with PIMD/IDs.

## Introduction

Profound or severe neuromotor dysfunctions, restricted or absence of limb functions, and severe or profound learning or intellectual disabilities (IDs) affect individuals with profound intellectual and multiple disabilities (PIMD) [1–5]. They often have comorbid complex functional or sensory impairments and chronic health conditions such as epilepsy, visual impairments, constipation, spasticity, deformations, and incontinence [6]. Their severe condition presents significant limitations in communication due to the inability to comprehend social cues, spoken, verbal or symbolic language [1–4]. These are often manifested in atypical, minute, and refined body and hand postures and movements, vocalizations and utterances, muscle tensions, or facial expressions on a presymbolic (nonsymbolic) or protosymbolic level with no shared meaning and stereotypical non-communicative behaviors [1–3,7–9]. Such extensive communication ambiguities impede expressing their needs, desires, thoughts, and feelings requiring great dependence on their close caregivers who are more capable of discerning and interpreting the most idiosyncratic behaviors than other people, thus limiting their communication group [2,7,10]. Supporting these individuals especially in communication is of crucial importance and this constitutes a great significance for studies which has gradually broadened from describing dialogical meaning-making, communication assessment approaches to the use of remote-controlled interactive objects that respond to gross body movement, focus of attention, and vocalizations [7,8,11]. A closer look at these studies reveals that the distinct and idiosyncratic behaviors and the individual differences are arguable of central interest in supporting communication especially among children with PIMD.

### AT, AI, and ML-based communication aids

Computer-based assistive technology (AT), artificial intelligence (AI), and machine learning (ML) algorithms have met great success in improving or aiding communication among children with neurological, genetic, motor, or physical impairments. Children with severe physical and speech impairments could improve eye gaze performance for communication and activities using gaze-based assistive technology (gaze-based AT) [12,13]. There is also evidence that eye-tracking technology is a potential satisfactory technology in supporting communication and psychosocial functioning of children with Rett syndrome who have speech and hand use impairments and severe motor apraxia [14]. Wearable-sensor-based platform systems consisting of gyroscopes, accelerometers, and global positioning system (GPS) have also been used to recognize the gesture movements of children with autism spectrum disorder (ASD) and communicate using several ML classification algorithms with mostly 91% accuracy rates [15].

The feasibility of camera-based body mapping and movements, computer vision, speech recognition technology, AI, and ML classification models have also been briefly introduced to support communication among children with PIMD [16]. The INSENSION project aimed to collect, extract, catalog, and interpret the behaviors or psychological states (pleasure, displeasure, or neutral) and their exhibited mode of communication such as protest, demand, or comment to provide feedback and propose what action or response the caregiver should take to enable them to communicate and control their surroundings [16]. Although this attempt is considered the first step towards a more profound understanding and interpreting the idiosyncratic behaviors and individual differences of children with PIMD/IDs, the published study presented insufficiently detailed methodological and analytic information especially on the classification of behaviors and psychological states using supervised or unsupervised ML algorithms and actual accuracy rates to allow replication and system operation.

### Prior work: Behavior data collection and categorization for ML modeling

To the extent of providing more insights, systematic and feasible approach in the uncharted area on the use of computer-based AT and ML to aid communication among children with PIMD/IDs, our current work focuses on developing a novel inference system that interprets their movements. This entails two major steps, the first of which is the collection, extraction, and initial categorization of the movements of the children based on the interpretations of their caregivers. This was initially performed using a previously developed mobile app called ChildSIDE that enabled the collection of 327 behavior data from 20 children-caregiver dyads [9]. Video-based retrospective inter-rater agreement Kappa analyses revealed that most of the movements of children with PIMD/IDs were manifested mainly through body (27.7%; approaching, contacting, and movement of part of the body) and hand movements (22.8%; pointing, reaching, and moving). This motivated the use of body and hand mapping and movement trajectory analyses using camera and movement analysis software. Thus, the second step is to compare the performances of several ML algorithms or classifiers to establish a stable and accurate behavior inference system.

### ML algorithms, dataset recalibration, binary and multiclass outcomes, and feature selection

Recent ML-based studies that aimed to classify or differentiate disorder sub-populations, distinguish behavioral phenotypes between and among disorders, and diagnosis, significantly targeted children with ASD, attention-deficit and/or hyperactivity disorder (AD/HD), internalizing disorders (anxiety and depression), down syndrome and paralyzed individuals [15,17–25]. Utilizing some of the most common and considered ML models are support vector machine (SVM), decision trees, random forest (RF), and neural network (NN), these studies have also looked in to comparing the variances in the classification performance of ML models, training recalibrating dataset combination, and classifying target behavior, movement or psychological and biological markers to multiclass behavior outcomes. Duda et al. (2016) compared the variances in the classification performances among six ML models such as decision tree, RF, SVM, logistic regression, categorical lasso, and linear discriminant analysis for the behavioral distinction of individuals with either ASD or AD/HD [19]. Their findings revealed that among the six, Support Vector Classification (SVC) performed with the highest accuracy of 96.5% which proved to be an optimal model in classification tasks with its low error rate and probabilistic qualities [19]. Further, comparing varied classification accuracy performance rates of ML models in training recalibrating dataset combination (using data from the previous or same session) and classifying multiclass behavior outcomes (3, 4, and 6 classes or reaching movements) were also investigated by Shiman and colleagues (2017). They aimed to classify several functioning reaching movements from the same limb using EEG oscillations in creating a more versatile brain-computer-interface (BCI) for rehabilitation of paralyzed patients [25]. Interesting results revealed a decreasing trend in the accuracy rates (67%, 62.7%, and 50.3%) in decoding three, four, and six task outcomes (or reaching movements from the same limb) respectively, which implies that using 3 classes could be used to control assistive or rehabilitation robotic devices for paralyzed patients [25]. In a non-human-behavior context, another proposed method in improving classification performance is the use of the Boruta feature selection method, especially when boosting the performance of the SVM classifier [26]. A simulation study systematically evaluated and compared the performances of variable selection approaches revealed that Boruta was the most powerful approach compared to other variable selection approaches like Altman, Permutation approach, recurrent relative variable importance or r2VIM, Recursive feature elimination or RFE, and Vita [27]. It has the most stable sensitivity in detecting causal variables while controlling the number of false-positive findings at a reasonable level compared with other state-of-the-art RF-based selection methods [28].

### App-based and sensor-collected location and weather data

ML-based behavior studies have sourced and analyzed behavior data from phenotypic, electrodermal activity (EDA), or virtual reality (VR) systems, wearable sensors, standardized scales and instruments or task or performance-based measures [15,17–19,21,23–25]. Mobile platforms and apps have also been validated using ML classifiers as a screening method, utilized to embed ML-based scoring models, and to deliver or collect the behavioral assessment, screening tools or data for ML-based analyses [9,29–32]. Collection of fine-grained and extensive records of behavioral data and expression across situations such as daily activities, social interactions and communication, mobility patterns, bodily functions, and biological markers, location (e.g. GPS map coordinates and iBeacon or BLE indoor), timestamps and logs have become possible using mobile devices [9,33–39]. Equipped with Wi-Fi, Bluetooth, near-field communication (NFC), and sensors harnessed by apps, smartphones have become a primary tool in extracting useful features with ML algorithms [40–44].

Human behavior metrics derived from apps and sensors, and location data have also proposed the use of weather variables in classifying user preferences and daily stress [43,45]. Developing several classification models from the users’ checkins (location-based social networking) based on different weather conditions (e.g. precipitation intensity, precipitation probability, apparent temperature, humidity, wind speed, wind bearing, visibility, and air pressure), ML classifiers obtain an average accuracy of 72.77% area under curve (AUC) in predicting users’ activity, venue, and transportation mode [43]. Weather variables (e.g. mean temperature, pressure, total precipitation, humidity, visibility, and wind speed) and Bluetooth proximity location features with behavioral metrics, derived from the user’s mobile phone activity (calls and SMS data), and personality traits have also been used to recognize daily stress. The inclusion of weather variables in the final model with other datasets provided a better accuracy rate (72.4%) for the recognition model for daily stress based on RF and gradient boost ML classifiers [45]. The consistent patterns of differences in weather variable indices associated with human activity and behaviors have been documented across settings and seasons and typically developing children in recent years [46]. These suggest sensitivity of behavior interventions to local weather conditions and potential weather impacts [47]. This study builds on this premise that location, weather indices and time features can be potentially used for ML-based classification of the movements of children with neurological and motor or physical impairments, more specifically, with PIMD/IDs which remains unexplored.

### Current work: ML-based behavior inference system

This study explores the ML-based framework structured on utilizing different classification models, recalibrated datasets, feature selection method, and the potential use of location, weather, and time data in developing a behavior inference computer-based communication-aid AT system to interpret the movements of children with PIMD/IDs. Using 53 features related to child movements and characteristics and location, time and weather indices, we investigated and compared the accuracy performances of ML classifiers (XGB, SVM, RF, and NN), feature selection method (Boruta), and recalibrating datasets (minor, major or both movement categories with or without environment data) on classifying the movements of children with PIMD/IDs to binary (2) or multiclass (3 and 7) behavior outcome classes. Further, the influences of recalibrated dataset, feature selection, classifiers, and classes on the pooled classification accuracy rates and the interactions among them were also evaluated. This study is exploratory on the inclusion of location, time, and weather indices as features, recalibrating datasets, selecting important features, and classifying the movements of children to binary and multiclass behavior outcomes using four ML models. However, we hypothesized that a high classification accuracy rate could be achieved in adding environment data to movement datasets and selecting important features using Boruta. Based on previous studies, we also hypothesized that the classification accuracy performance would be higher in classifying binary behavior outcomes using RF and SVM-based classifiers for a stable behavior inference system. The contributions of our work are stated below:

- We are proponents of the underexplored yet innovative use of sensor-or-app-collected location, time, and weather indices on developing assistive technology programs (apps, devices, and behavior inference system) to support children with communication, physical or other neurodevelopmental disorders. As evident from our results, environment data can be important features on classifying the movements of children with PIMD/IDs.
- Our previous [9,39] and current work are aligned to the aim of supporting children with communication, physical or other impairments, specifically the underrepresented population of children with PIMD/IDs, to have independent communication and mobility. For such purpose, we introduce a behavior inference computer-based communication-aid AT system to interpret the movements of children with PIMD/IDs. It is a viable solution that embeds ML framework incorporating motion capture and gesture recognition, app-based voice output communication aid (VOCA) and home-appliance-connected smart speakers to respond to the children’s behaviors, needs or desires or aid caregiver support.
- Our work presents one of the first investigations on structuring behavior and communication interventions to ML framework to an unchartered area of aiding the communication of children with PIMD/IDs. Moreover, our study also proposes a considerably new approach, an interesting ensemble of several ML approaches (dataset recalibration, feature selection, and ML classifiers, binary and multiclass behavior outcome classes) to obtain high classification performance rates. Rather than solely relying on the power of a specific ML model, indeed, applying dataset recalibration (adding environment data to movement categories datasets), selecting important features using Boruta (>70% of the 53 baseline features are considered important), using the most common and efficient ML models (RF, SV, and NN), and classifying movements to binary or multiclass (3) behavior outcome classes, provided relatively high classification accuracy rates of >73%.

In this present work, we propose the interpretation of the movements of children with PIMD/IDs structured on several ML-based approaches (dataset recalibration, feature selection, and ML classifiers, binary and multiclass behavior outcome classes) by collecting child characteristics and movements with simultaneous environment data which are illustrated and described in the methods section. The statistical analysis for classification accuracy rates comparisons is also described in the materials and methods section. The results section presents the important features selected by Boruta and the variances in the classification accuracy rates by dataset recalibration, class, movement dataset with or without environment data, with or without feature selection training, and classifiers. In addition, analyses of variances evaluating the interactions among behavior outcome classes, dataset recalibration, feature selection, and ML classifiers and their influences on the mean classification accuracy rates are also described. The main results were discussed and explained by or compared to related previous investigations in the discussion section. Lastly, conclusions were drawn based on the hypotheses and structured based on the main results.

## Materials and methods

### Participants, sessions and experimental setup

**Fig 1:**
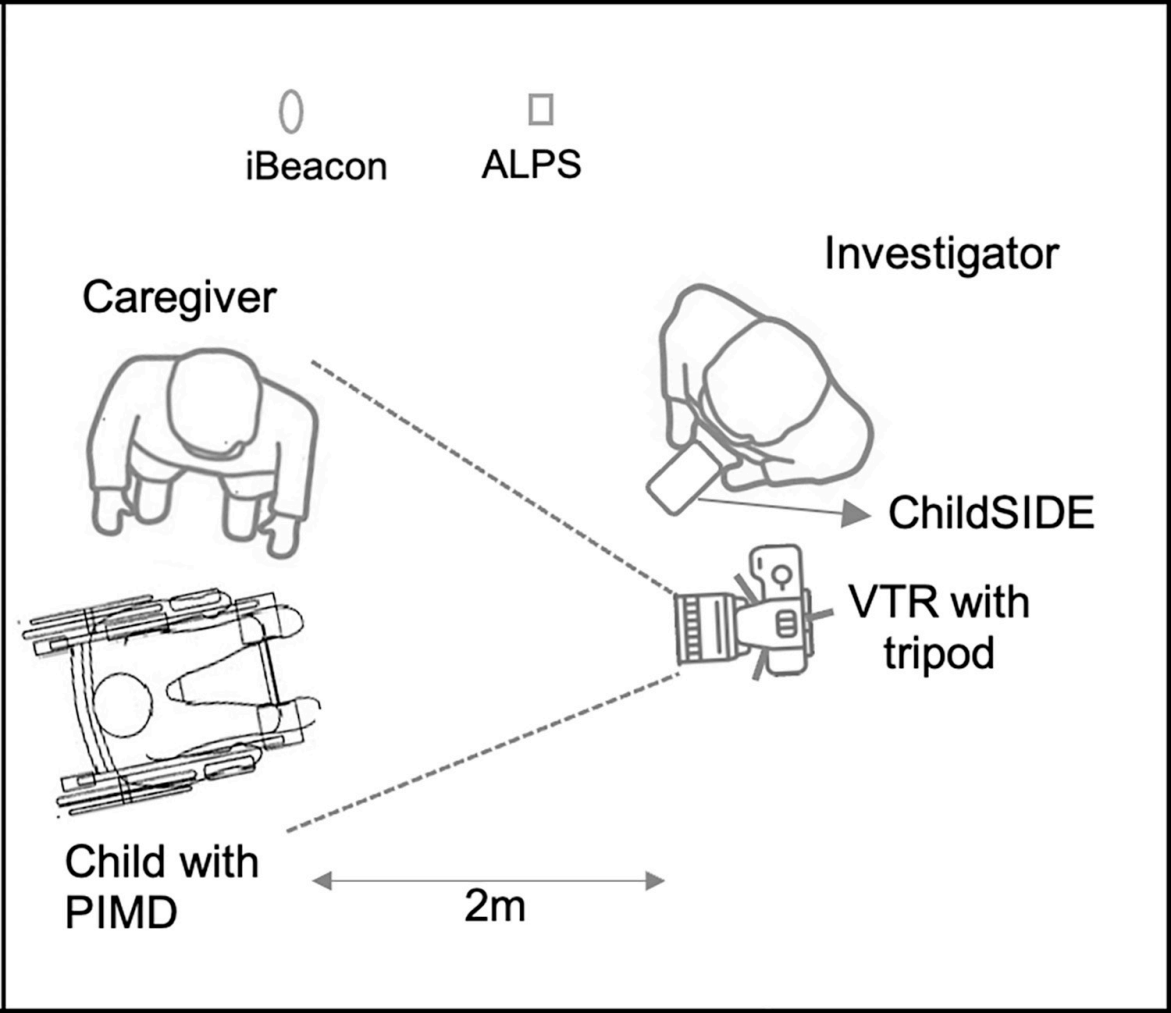
Experimental set-up showing the location and weather sensors and the videotape recorder (VTR) placed 2 meters from child with PIMD/IDs and caregiver.

A total of 105 (ranged from 1 to 15 sessions per dyad) single-dyad face-to-face and video-recorded sessions (30-hour; average:18.5 mins.; recording time ranged from 0.37 to 54 mins.) were conducted among 20 purposively sampled children whose ages were from eight to 16 years old (3rd grade to 1st-year high school), who were mostly males (68%) and had either PIMD (n = 15; 79%) or severe or profound IDs (n = 4; 21%) and their caregivers. Natural-child-caregiver-dyadic interactions were observed targeting facial, upper and lower body and limb movements during morning greetings, lunchtime, and break time in different locations within their school. One investigator recorded all the caregiver-interpreted movements using an app connected to a multi-function weather sensor device, online weather API, GPS and proximity sensing device and timestamp, which were simultaneously collected with each individual movement data [9]. Our experimental setup (Fig 1) shows the videotape recorder (VTR) was placed 2 meters from the subjects. The movements were then categorized retrospectively (inter-rater agreement Kappa analyses) based mainly on the outputs (movements and behavior matrix and interaction-based theory of attuning) of previous observational studies among children with PIMD/IDs [9,48,49]. This study was written, approved, and performed as per international ethical guidelines (ethics approval number of the project: R2-18) [50,51]. Signed written informed consents were obtained from the caregivers or parents who were also informed that their participation in the study was voluntary and that they may stop their participation at any time. Forms, data or any identifiable information that may reveal participant identity were coded and stored in a password-protected network server database and computer for protection and privacy.

### Data analyses workflow

**Fig 2:**
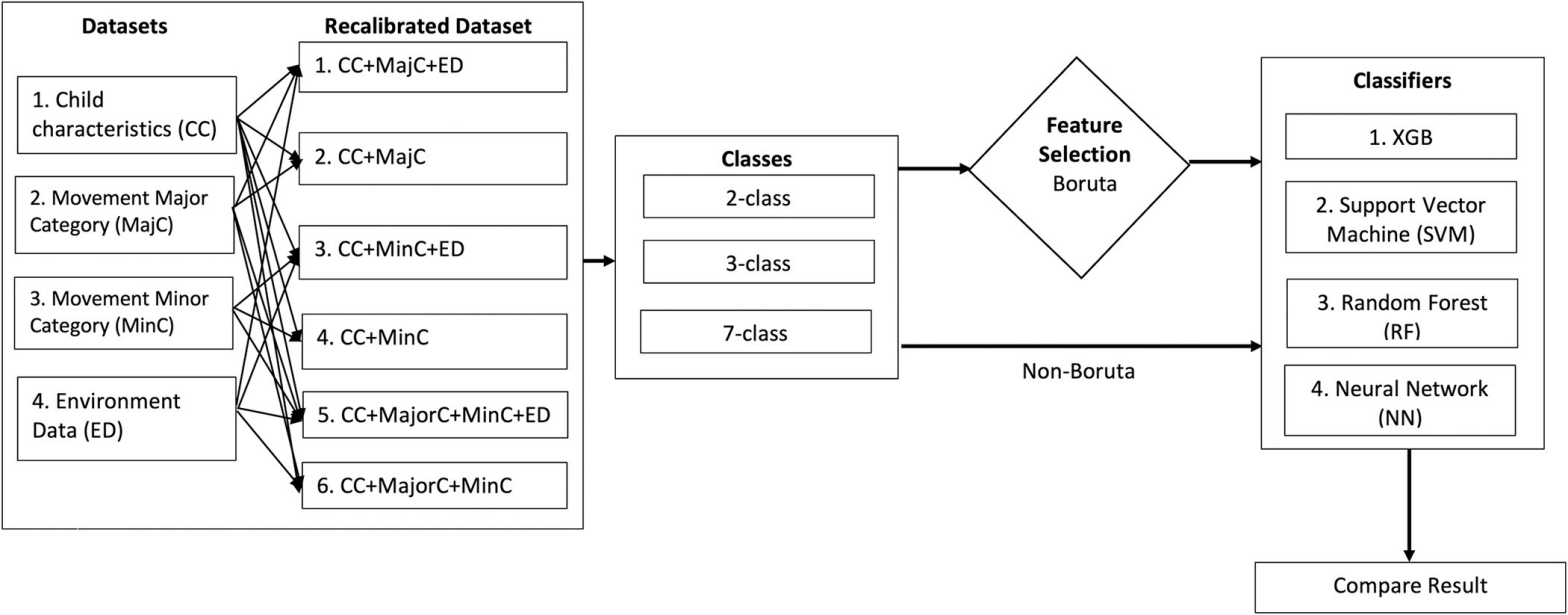
Data analyses workflow from dataset combination to classification accuracy comparison.

Fig 2 illustrates the workflow of the data ML analyses. Our datasets consisted of 53 baseline features related to the characteristics (gender and condition) of the children with PIMD/IDs (CC), their movements that were categorized into 5 major (MajC) and 10 minor (MinC) categories, and 36 environment data (ED) consisted of time, location and weather indices. To investigate whether recalibrating the dataset with the inclusion of ED would allow a more accurate classification of either major or minor movement categories, we made several combinations. Each dataset combination has child characteristics, with major or minor (or both) movement categories with or without ED which were trained to classify movements to 2, 3, and 7 behavior outcome classes. The recalibrated dataset combinations in each class were subjected to feature selection (using Boruta) or not (non-Boruta) to investigate whether the feature selection method will improve the computational speed and accuracy of four classifiers which were primarily compared based on accuracy rates. In total, we evaluated and compared the classification accuracy rates among 48 patterns by recalibrated dataset (6), with and without Boruta feature selection method (2), and ML classifiers (4). This was done using R (version 4.0.3) free software programming language [52].

### Features

#### Child characteristics

All recalibrated movement dataset included children’s gender (male or female) and their main condition as features. The children included in this study had main diagnoses of either profound intellectual and multiple disabilities (PIMD) or severe or profound intellectual disabilities (IDs).

#### Major and minor movement categories

Using retrospective inter-agreement Kappa statistics analyses, 676 upper and lower body and limb movements extracted from 291 individual movement and behavior data were categorized into major and minor categories [9]. In this study, movement features were composed of 5 major and 10 minor categories [9]. *Eye movement* included gazing (at people and things especially unfamiliar faces), and changing line of sight (gaze rolls and moves, point-like tracking, momentary glares) minor categories. Movements that involved smiling, and showing facial expressions (surprised, frowning, sticking out tongue, other than smile) were categorized under *facial expression* major category. Vocalization was considered as a major and as a minor category. Pointing (hand or finger towards an object) the action of reaching (or chasing after reaching the target, not by pointing hand or finger), and moving hands (for grabbing, hitting, pushing, or raising) were categorized as *hand movements*. *Body movements* comprised of approaching movements using head or upper or whole body (moving close to a person or an object), and movement of any part of the upper body including head and neck, and upper or lower limbs (shaking, bending, moving mouth, etc.). Pre-processing for classification involved the conversion of major and minor categories to numerical data using integer and one-hot encoding.

#### Environment data (location, time, and weather) and k-NN imputation

Location (4 outdoor GPS and iBeacon indoor location proximity measurements) and weather indices (11 ALPS sensor: UV, 6-axis (Acceleration + Geomagnetism), ambient light, atmospheric pressure, temperature, humidity, and; 8 OpenWeatherMap API indices: minimum, maximum, and main temperature, atmospheric pressure, humidity, cloudiness, wind direction, and wind speed) that were collected simultaneously with the movements data, comprised the 23 ED features. We have also included 13 features derived from the timestamps (seasons: autumn and winter, date: years, months, day, time: hours, minutes, and seconds) in ED.

In cases where there was only a single missing value in a dataset collected in a specific location and time within a session, the values of the nearest non-missing data were used to identify the possible value of that missing data. For instance, a missing iBeacon data should be similar to the non-missing iBeacon data collected within the same session at the same video time frame and location. However, in cases when there were multiple missing data and identifying which nearest non-missing data to input in the missing data, our preprocessing involved k-Nearest Neighbor (k-NN) imputation (k = 14). This was done to avoid relatively large average errors due to the anticipated decrease in the size of our dataset after deleting individual behavior data with no matched ED [53]. k-NN impute is the KNN algorithm’s function to impute the missing data values, which, compared to deleting the rows with missing data, can easily and effectively handle a number of missing values in large datasets [53]. This technique identifies the partial feature in a dataset and selects the K nearest data (by calculating the distance) from training data with the known values to be imputed. Then it substitutes the missing value with an estimated value (using the mean) of the data with known or non-missing data values [53]. Pre-processing also involved transforming categorical data (e.g. iBeacon name, seasons, days of the week, years, and months) to continuous data using integer and one-hot encoding.

### Binary and multiclass behavior outcome classes (Attuning theory)

Griffiths and Smith (2017) discussed in length how people with severe or PIMD communicate with others by introducing Attuning Theory [49]. This theory suggests that the communication between individuals with PIMD and their caregivers is regulated by the process of attuning which describes how they move towards or away from each other cognitively and affectively [49]. This core category consists of seven discrete but dynamically interrelated categories namely: *setting*, *being*, *stimulus*, *attention*, *action* (including maneuvering), *engagement*, *and attuning*. The proponents described and connected each category in a synopsis where all communication occurs in an environment or “setting” which is described as the total context of a place (e.g. bus, field, kitchen, etc.) which influences the individuals’ feelings and their state of mind (their being) or their action [49]. The “being” influences how each person behaves which is the stimulus that impacts the way people attend to each other (attention) and the nature of the interaction (engagement). Whether or not an “action” or engagement actually occurs is determined by the process of attuning which affects and reflects how the individuals perceive or feel their state of being and deliver stimulus to each other. Further, they comprehensively described in detail the structure (anti and pro and negative and positive), typology (from screaming to harmony), indicators (looking at each other, movement towards each other, smile, close physical contact, gaze, expression, etc.) and codes (concentration, interest, and support) of attuning. Griffiths also listed and described the behavior and movement manifestations of each category (e.g. attention is manifested by visual tracking, mobile gaze changes, still gaze, head position, etc.) [49].

An inter-rater agreement Kappa statistics analysis was conducted by four independent behavior expert raters to interpret and classify the 291 individual movement data to binary (2), 3 and 7 behavior outcome classes. Each individual movement data was analyzed and coded with numbers that correspond to a class. Initially, we grouped the movement data into 9 behavior outcomes (first outcome level): “calling”, “response”, “emotions”, “interest”, “negative”, “selecting”, “physiological response”, “positive” and “unknown interpretations” (Table 1). The behavior outcome classes that we created were similar to that of Attuning Theory’s indicators, codes, or categories like *engagement (joint attention)*, *assent*, *harmony*, *delight*, *please or pleasure*, *interest*, *and pro and negative attuning (refusal)* [49]. However, we also developed new ones as required (“selecting” and “physiological response” categories). From the 9 behavior outcome classes, we had to delete the “unknown” and “positive” categories due to lack and difficulty in identifying operational definitions from the extracted movements and due to a small sample sizes.

**Table 1:** Movement definitions and manifestations of the 7 behavior outcome classes in comparison with the Attuning Theory.

This study			Attuning Theory	
Category	Definition	Manifestations (sample extracts)	Category	Definition
Calling	Verbal (e.g. greetings, vocalization) or non-verbal behavior (e.g. smile, staring, pointing, etc.) aim to get the attention of the caregiver or teacher	-moves mouth to say only the "mas" part of "Ohayo gozaimasu"; -Move the face widely. Open mouth wide and try to speak. Breathe a little harder; -Vocalizes while touching the back of the neck with the left hand; -Looks out of the window at the car and says "Densha" (street car); -Pats the teacher on the back. Turning to face her and mumbling something.	Engagement (joint attention)	Engagement of both partners in the dyad may be directed to the same focus.
Response	Verbal (e.g. “yes”, “bye-bye”, etc.) or non-verbal (e.g. raises hand, nodding, wave hands, clapping, etc.) responses to other’s questions or gives signals to other person	-Pointing or pushing somewhere in the book with the left hand; -Looks at the teacher’s face and says "yes". Makes a slight nodding movement; -Raises both arms upwards. Raising the corners of the mouth. Saying "mmm". Shake your head from side to side. Shake head vertically. Movement of the mouth. -Vocalization. Moving the body. Increased breathing. Eye movement.	Assent	demonstrates attuned agreement between the dyad. One partner carries out an action or asks a question and the other responds in a clear affirmative manner.
Emotions	Mostly non-verbal expressions of feelings of being happy, pleasure, excited, perception of fun, angry, worried, troubled etc. (e.g. smile, moving or opening mouth, shaking head vertically or body, looking away, etc.)	**-**being delighted, raises the corner of his mouth and shakes his face from side to side while holding the back of his head with his right hand; -Pleasant feeling, open mouth and bring hand to mouth. Looks at the teacher and opens eyes. Raises eyebrows upwards; -feeling angry, body begins to sway. The corners of the mouth and the corners of the eyebrows fall. Suddenly stands up and walks over to the TV; -feeling troubled, move the right hand up and down in front of the face. Say the words Move the right hand back and forth. Touching the front teeth with the left hand.	Harmony, delight, pleased, pleasure	*Harmony*, characterized by actions and communications that display mutual satisfaction. *Pleased* demonstrates with smiles, grins and other visual appearances of satisfaction, a quiet contentment with what is going on. This differs from *pleasure*, describing a more intense satisfaction, where smiling veers towards laughter, where the communication is more intense and direct.
Interest	Verbal (e.g. “let me see”, “yes!”, “what’s that?”) or non-verbal (e.e.g pointing, raising hands, standing up, nodding, etc.) that hints interest in an object, person or action or doing an action.	-Opens mouth wide, smiles and bends over. Says "Oh, hi, hi, hi" in a strained voice; -Stares at a ball. Moves towards the toy; -Pointing and saying "Oh, what?"; -Stands up and walks to the teacher in front of him; -Says "woo". Touches front teeth with left hand. Moves left hand to right ear Eye movement. Mouth movement Repeated raising and lowering of the right hand (in front of the face). Looks down; -Smiling and nodding.	Interest	the communication partner demonstrates an obvious attention and interest in (attuning to) the action that is going on. The attention is focused through the action. The result of the interplay of attention and action is that the attuning level of the partners rises and falls in tandem with the attention displayed to the action.
Negative	Verbal (e.g. “no”, “don’t like”, “dislike” or “end”) or non-verbal actions and vocalizations (e.g. closes mouth, sticks out tongue, turns face away) to express refusal or disagreement.	-refuses to take a spoonful of rice in his mouth. Closes his mouth when a spoon is put close to his mouth; -pushes away teacher’s hand. Closes mouth. Slaps own body with hand; -frowns and pushes the teacher’s body with his right hand; -touches his face (mouth and nose) with his hands while moving his fingers.	Pro and negative attuning (Refusal)	In this state, pro attuning coexists with negative attuning. The communication partners understand each other very well (high pro-attuning). However, they do not accede to the wishes of the other so the interplay between the dyad is negative.
Selecting	Mostly non-verbal actions or gestures (e.g. pointing, tapping, reaching) to express decision or desire to choose between or among objects.	-Points to a picture book. Says a sound similar to "this"; -Looks around at the side dishes and selects a side dish by saying "this one" with the index finger of the left hand; -Looks at what the teacher is pointing at. Moves left hand; -Tapping the teacher’s/caregiver’s foot; -Points to the numbers on the board with hand.	-	-
Physiological response	Verbal (e.g. saying “rice”, “sleepy”, “thirsty”, etc.) and non-verbal (e.g. closing eyes, not opening mouth) vocalizations and actions to express functions or desires relating to normal physical or bodily responses.	-sleepy, eyelids close. Look up and do not move; -sleepy, mouth opening is too small. Refuses to take food in mouth; -thirsty, calls for the caregiver/teacher three times. -sleepy, looks down. Movement becomes stiff; -in pain, frowning and touching the teacher’s hand with the right hand; -tired, plops down on desk. Sneezing. -says "rice" while looking at the table.	-	-

**Fig 3:**
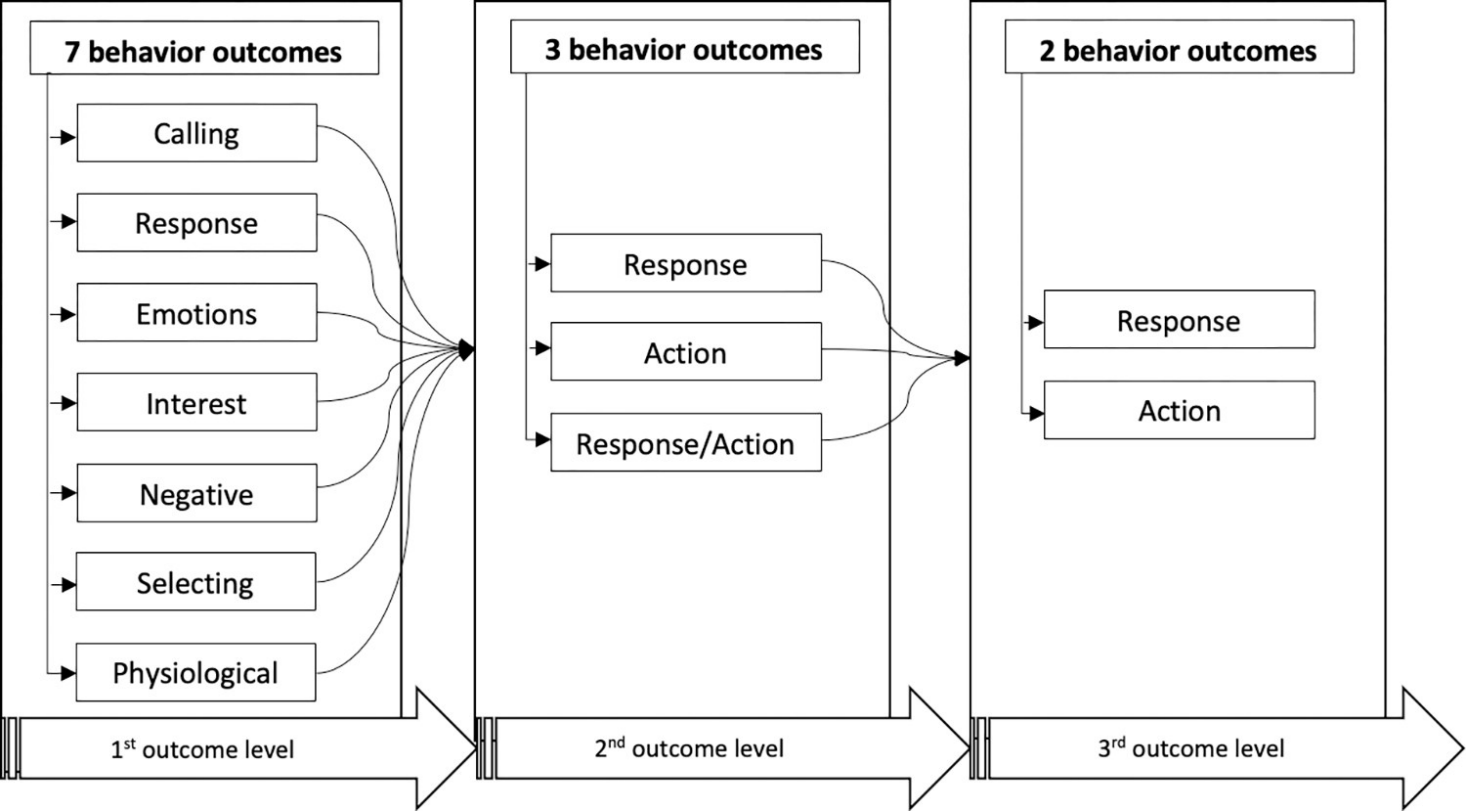
Behavior outcome classes in each outcome level.

The individual movement data were then analyzed and grouped into 3 behavior outcomes (2nd outcome level) which are “response”, “action”, and “response or action” (Table 2), similar in partial with Attuning theory’s stimulus (non-action), action (dual response) and the relationship between stimulus and action category definitions [49]. In the last level, the third behavior outcomes, the individual behavior data were categorized as either “response” or “action” using the definition used in the 2nd outcome level (Fig 3). In each outcome level, Kappa statistics were computed to identify the level of agreement between and among the experts. In cases when there were low agreement levels (1.01 to 0.60), a series of pair (first and second outcome levels) and group (third outcome level) expert brainstorming sessions were conducted until an acceptable to almost perfect agreement levels (0.61 to 0.99) were reached before proceeding to another outcome level.

**Table 2:** Feature (category) description of the 3 and 2 behavior outcome classes in comparison with the Attuning Theory.

This study				Attuning Theory	
Outcome classes		Category	Definition	Category	Definition
3 behavior outcome classes	2 behavior outcome classes	Response	a one-way communication (from the perspective of the caregiver/teacher) that stimulus from the child (movements, gestures, facial expressions, vocalization or other behavior) may affect or influence the attention of the caregiver or teacher but don’t necessary require an action response from the caregiver/teacher.	Stimulus (Non-action)	Stimulus is an attempt by one partner to encourage an action from another partner. Non-action is concerned with settings where minimal stimuli are present, but no action is elicited from the participant (s). It may be passive or active (determined inaction) or occur as result of a period of stasis.
		Action	two-way or mutual communication (from the perspective of the caregiver/teacher) where the stimulus from the child (movements, gestures, facial expressions, vocalization or other behavior) affects or influence the attention of the caregiver or teacher which cause a response through action (e.g. attending to children’s needs).	Action (Dual action)	Actions are observable process of behavioral change in an individual that is demonstrated by movement, gestures, facial expression, vocalization or other behaviors. It can be a dual action where action may be carried out by both participants in the dyad, that is, they may work together to achieve an action. Dual action arises where one participant carries out part of an action, but the other completes it.
	Response/Action		stimulus from the child (verbal or non-verbal responses or behavior manifestations through movements, gestures, facial expressions, vocalization or other behavior) which affect or influence the attention of the caregiver or teacher which may or may not require responding through action.	*“The dividing line between these concepts is that a code is grouped under action if it comes about as a result of a previous stimulus or is to be an event that is not designed to elicit a reaction*, *whereas a code is grouped under stimulus if it clear that a stimulus is provided in order to induce a certain course of action*.*”*	

### Feature selection (Boruta)

Feature selection identifies important features useful for model prediction from the randomly expanded system produced by merging the randomly permuted features and the original training features [28]. To assess the importance of the feature in the original system, Z-scores of the original features and the randomly permuted features were compared, in which the maximum Z-scores among the randomly permuted features were identified [28]. If the Z-score of the feature was higher than that of the maximum Z-scores among the randomly permuted features, the feature was deemed important (confirmed), otherwise, not important (rejected). For each run, the randomly permuted data was different. Using maximal importance of the randomly permuted features, a two-sided binomial test identifies an important feature if the number of times it was found important (observed) was significantly higher than the expected number (0.5N) at significance level a, otherwise, will be excluded from the system in subsequent runs. We implemented it in the R package Boruta which uses an RF method using the maxRuns parameter which will force the run to stop prematurely. The features that were neither important nor unimportant were tagged as tentative. In this study, as recommended by default, we set the confidence level a = 0.01 and run the algorithm with 100 maxRuns due to fewer features were included in the model.

### ML classifiers

We tested the classification performances of XGB, SVM, RF, and NN classifiers on our dataset combinations (major and minor behavior categories with and without environment data) trained with or without feature selection (Boruta) to conduct binary and multiclass outcome behavior classes. Dataset has been partitioned into two parts (training and testing) where 80% were validation data which were tested with 20% remaining data to tune for hyperparameters. The stratified extraction method was used to avoid bias in dividing the data. To avoid the problem of overfitting and underfitting, 10-fold cross-validation was done. The selection of the classifiers included in this investigation were the most common and considered classifiers from previous behavior studies particularly SVM, RF, and NN. Considered as a powerful method for classification which creates hyperplane (maximum of p-1 planes) between classes (datasets with same properties considered as p-dimensional vectors) to predict labels from support vectors, SVM attempts to identify the best classifier/hyperplane which is located in the middle which has the maximum margin between the two classes (maximum-margin hyper-plane). Another algorithm that was designed to build several decision or classification trees that are consolidated to produce a precise and stable prediction is RF. In this model, each tree is constructed using different “bootstrap” or the same number of randomly selected samples from the original sample data used as its replacement. From this random sample, an estimated 63% of the original sample occur at least once and the remaining 1/3 was not used to build a tree and instead used for performing out-of-bag error estimate and feature importance. Then it randomly selects data nodes to construct a decision tree. Another classifier, NN is consist of nodes of artificial neurons which are represented by some state (0 or 1) or weight assigned to them to define its importance in each layer, from input, middle (hidden), an output layer, and the desired outcome that is more predictive of the specified outcome. The number of classes in multiclass classification commonly corresponds to the number of nodes in the outer layer. In addition, we also included a classifier that has robust power to control overfitting which extends the Boosting Tree models by performing second-order Taylor expansion of the objective functions after each split and adds splitting threshold (except if the information gain is greater than the threshold). The classification accuracy performance rates of all four ML classifiers were compared by accuracy, precision, recall or sensitivity, specificity, the area under the curve (AUC). Accuracy indicates how the classifier is often correct in the diagnosis of whether the major or minor movement categories are better with ED or not, while precision has been used to determine the classifier’s ability to provide a correct positive classification of the movements. Recall or sensitivity and specificity were used to identify the proportion of actual movements correctly identified by the classifier and to determine the classifier’s capability of determining negative cases of movements, respectively. The average AUC was also used to assess the goodness of a classifier’s performance which resulted from the 10 cross-validation trials where a value near 1 is termed as the optimal classifier.

### Statistical analysis

Pre-comparison, the classification accuracy rates (%) of the 10-fold cross-validation results were averaged to obtain the mean classification accuracy rates. First, to identify the recalibrated dataset combination with the highest accuracy rate (%), we conducted: a) a multi-stage comparison using one-way ANOVA (2-tailed) with Bonferroni *posthoc* test for multicategory variables, and independent t-test for binary variables (S1 File), b) using the same mean comparison analyses, the mean classification accuracy rate of each recalibrated dataset combination (a-f) was compared within (class 2, class 3, and class 7) and between classes (similar recalibrated dataset combination were compared across the classes), and c) the mean classification accuracy rates were then compared by recalibrated dataset in each class. Second, the pooled mean classification accuracy rates were compared by: a) class (2, 3 and 7 classes), b) ED inclusion (with or without ED), c) feature selection (with and without Boruta), and d) classifiers (XGB, SVM, RF, NN) in each class.

We used the univariate General Linear Model (GLM) feature of SPSS to conduct a three-way ANOVA to analyze the influences of and the interaction among the four factors (recalibrated datasets, feature selection, classifiers and classes) on the pooled mean classification accuracy rate (dependent variable). The factors were coded as categorical independent variables: recalibrated dataset (with ED “1”, without ED “0”), feature selection (with Boruta “1”, without Boruta “0”), and classifiers (XGB: “1”, SVM: “2”, RF: “3”, NN: “4”), and classes (class 2: “1”, class 3: “2” and, class 7: “3”). The degree of the influences of the four factors on the pooled mean classification accuracy rates and the interactions among them was identified using the partial eta squared (η^2^) and a subsequent Bonferroni *posthoc* tests were performed when the influences of the factors and their interactions on the pooled mean classification accuracy rate reached significance (p < .05).

## Results

### 1. Recalibrated datasets

1.a. The significantly highest classification accuracy rates were found in the Boruta-trained CC+MinC+ED dataset using the NN model in class 2 (76.33%) and the non-Boruta CC+MinC+ED dataset using SVM classifier in class 3 (76.3%) (S2 File).

**Fig 4:**
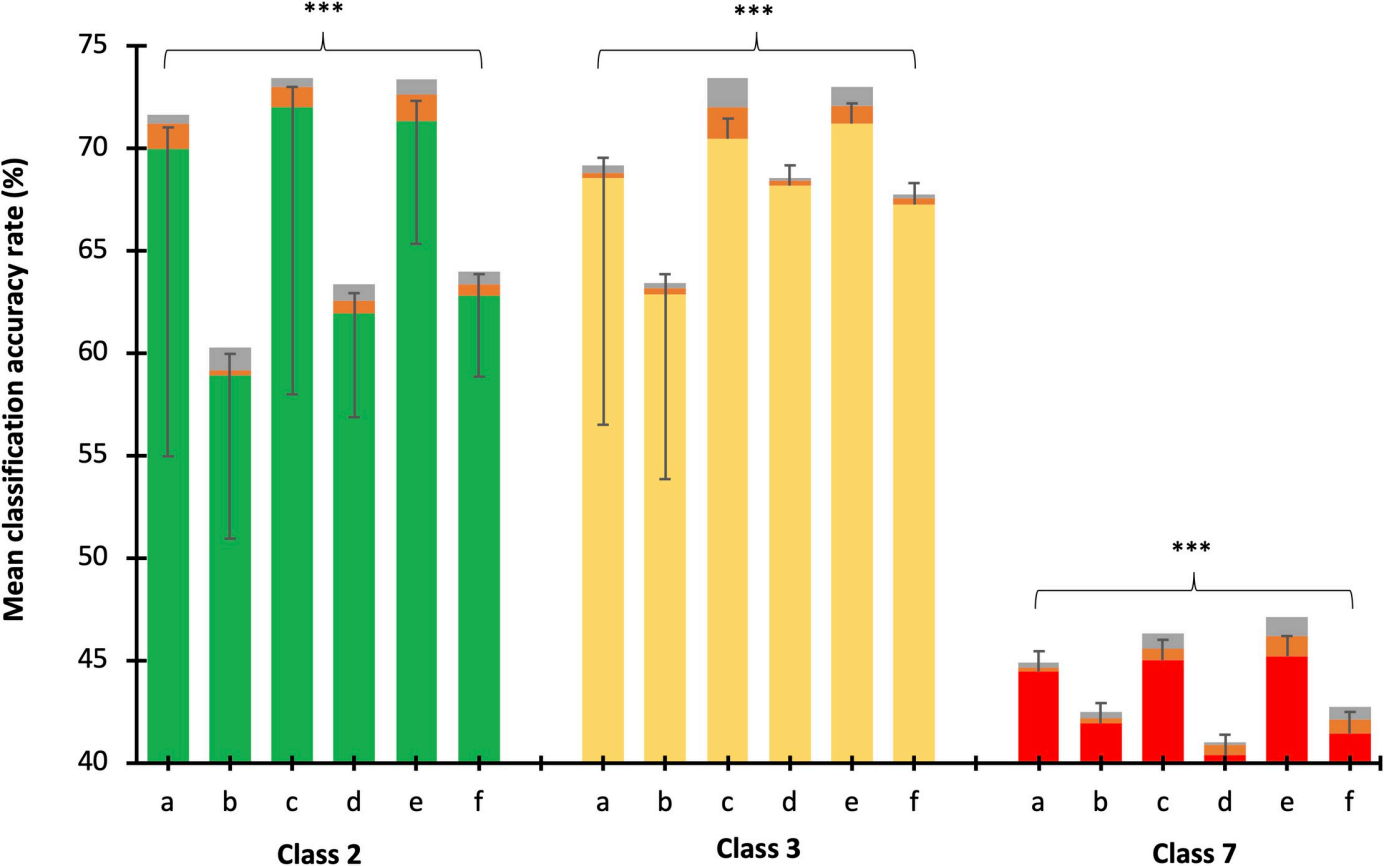
Mean classification accuracy rates (%) of each recalibrated dataset combination (a-f) within and between class comparison using one-way ANOVA (2-tailed) with Bonferroni *posthoc* test. a = child characteristics with major movement category and environment data (CC+MajC+ED); b = child characteristics and major movement category (CC+MajC); c = child characteristics with minor movement category and environment data (CC+MinC+ED); d = child characteristics and minor movement category (CC+MinC); e = child characteristics with major and minor movement categories and environment data (CC+MajC+MinC+ED); f = child characteristics with major and minor movement categories (CC+MajC+MinC); p < 0.001***, p < 0.01**, p < 0.05*.

1.b. One-way ANOVA revealed that the recalibrated datasets with the significantly (P < .001) highest mean classification accuracy rates were CC+MajC+ED (a) (70.4%), CC+MinC+ED (c) (72.4%), and CC+MajC+MinC+ED (e) (72.1%) in class 2, class 3 (68.9%, 71.9%, 72.1%, respectively; p < 0.001), and in class 7 (44.8%, 45.8%, and 46.2%, respectively; p < 0.001) as shown in Fig 4. When we compared the dataset combinations with the highest accuracy rates among classes, datasets a, c, and e of classes 2 and 3 had the highest accuracy rates among all the recalibrated dataset combinations (p < 0.001 *posthoc* Bonferroni test).

1.c. In classifying binary behavior outcomes (“response” versus “action”), the highest mean classification accuracy rates were obtained by the Boruta-trained dataset with ED using NN classifier (74.7%) (Table 3 in the left). The highest mean classification accuracy rate for classifying 3 behavior outcomes (“response”, “action”, and “response or action”) was with the non-Boruta-trained dataset with ED using SVM (73.5%), and RF (73.3%) (Table 3 in the middle). Non-Boruta dataset with ED in RF classifier had the highest mean classification accuracy rate of 47.1% in classifying 7 behavior outcomes (“calling”, “emotion”, “interest”, “negative”, “physical response”, “response”, and “selection”).

**Table 3:** The classification accuracy rate by recalibrated dataset (with and without environment data) in each class.

	2-class					3-class					7-class				
	(+) ED		(-) ED		Acc.	(+) ED		(-) ED		Acc.	(+) ED		(-) ED		Acc.
	(+)Bor	(-)Bor	(+)Bor	(-)Bor	Mean (SD)	(+)Bor	(-)Bor	(+)Bor	(-)Bor	Mean (SD)	(+)Bor	(-)Bor	(+)Bor	(-)Bor	Mean (SD)
XGB	69.03	67.60	59.10	64.37	65.03 (4.39)	69.27	69.63	65.77	66.23	67.73 (2.00)	43.80	46.73	39.63	43.20	43.34 (2.92)
SVM	72.13	72.03	59.07	62.90	66.53 (6.57)	70.67	73.50	65.43	67.17	69.19 (3.61)	46.53	45.07	38.63	43.10	43.33 (3.45)
RF	71.63	73.33	59.53	63.37	66.97 (6.57)	70.93	73.30	65.60	66.90	69.18 (3.56)	46.57	47.07	41.20	42.77	44.40 (2.88)
NN	74.73	72.57	61.87	66.33	68.88 (5.86)	69.60	70.93	65.47	67.83	68.46 (2.34)	44.47	44.27	41.33	43.07	43.29 (1.47)
Mean (SD)	71.88 (2.34)	71.38 (2.57)	59.89 (1.35)	64.24 (1.50)	66.84 (1.59)	70.12 (0.79)	71.84 (1.90)	65.57 (0.17)	67.03 (0.67)	68.63 (0.70)	45.34 (1.42)	45.79 (1.32)	40.20 (1.31)	43.04 (0.17)	43.60 (0.55)

Note: (+) ED = dataset with environment data; (-) ED = dataset with environment data; Acc. = accuracy; (+) Bor = with Boruta feature selection; (-) Bor = without Boruta feature selection; XGB = eXtreme Gradient Boosting; SVM = support vector machine; RF = random forest; NN = neural network; SD = standard deviation.

### 2. Pooled classification rates by ED, feature selection, and classifier

#### 2.a. Class

**Fig 5:**
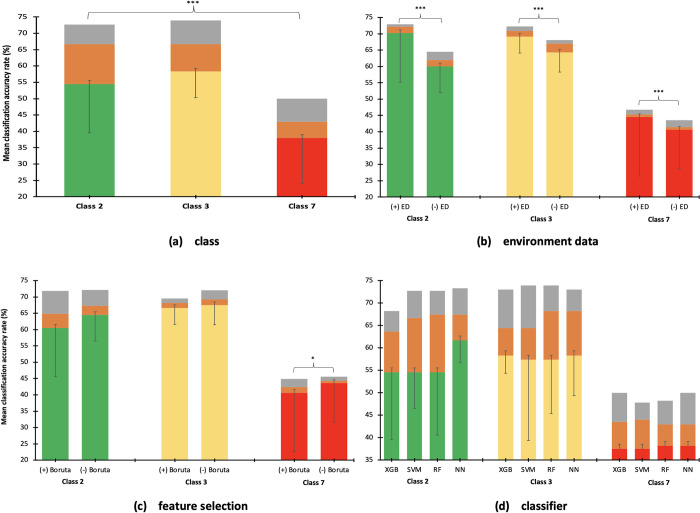
Mean classification accuracy rates (%) comparison by class, inclusion of environment data, feature selection and classifier. (+) ED = dataset with environment data; (-) ED = dataset with environment data; (+) Bor = with Boruta feature selection; (-) Bor = without Boruta feature selection; XGB = eXtreme Gradient Boosting; SVM = support vector machine; RF = random forest; NN = neural network.

The mean classification accuracy rates of each class were significantly different, where the highest was obtained by class 3 (68.63%; SD = 0.70), followed by class 2 (66.8%; SD = 1.6), and class7 (43.6%; SD = 0.55) (p < 0.001) (Fig 5A).

#### 2.b. Environment data

In class 2, movement datasets with ED (71.6%) were significantly higher than datasets without ED (62.1%, P < .001) (Fig 5B). Significantly higher classification accuracy rates were also obtained by the datasets with ED (71%) compared with datasets without ED (66.3%, p < 0.001) in class 3 and class 7 (45.6%, 41.6%, respectively) (p < 0.001).

#### 2.c. Feature selection

In all behavior outcome classes, the mean classification accuracy rates of the datasets which were not trained with Boruta (class 2: 67.8%, class 3: 69.4%, and class 7: 44.4%) were higher than Boruta-trained datasets (class 2: 65.9%, class 3: 67.83%, class 7: 42.8%). However, statistically significant differences were only found in class 7 (P = 0.03) (Fig 5C).

**Fig 6:**
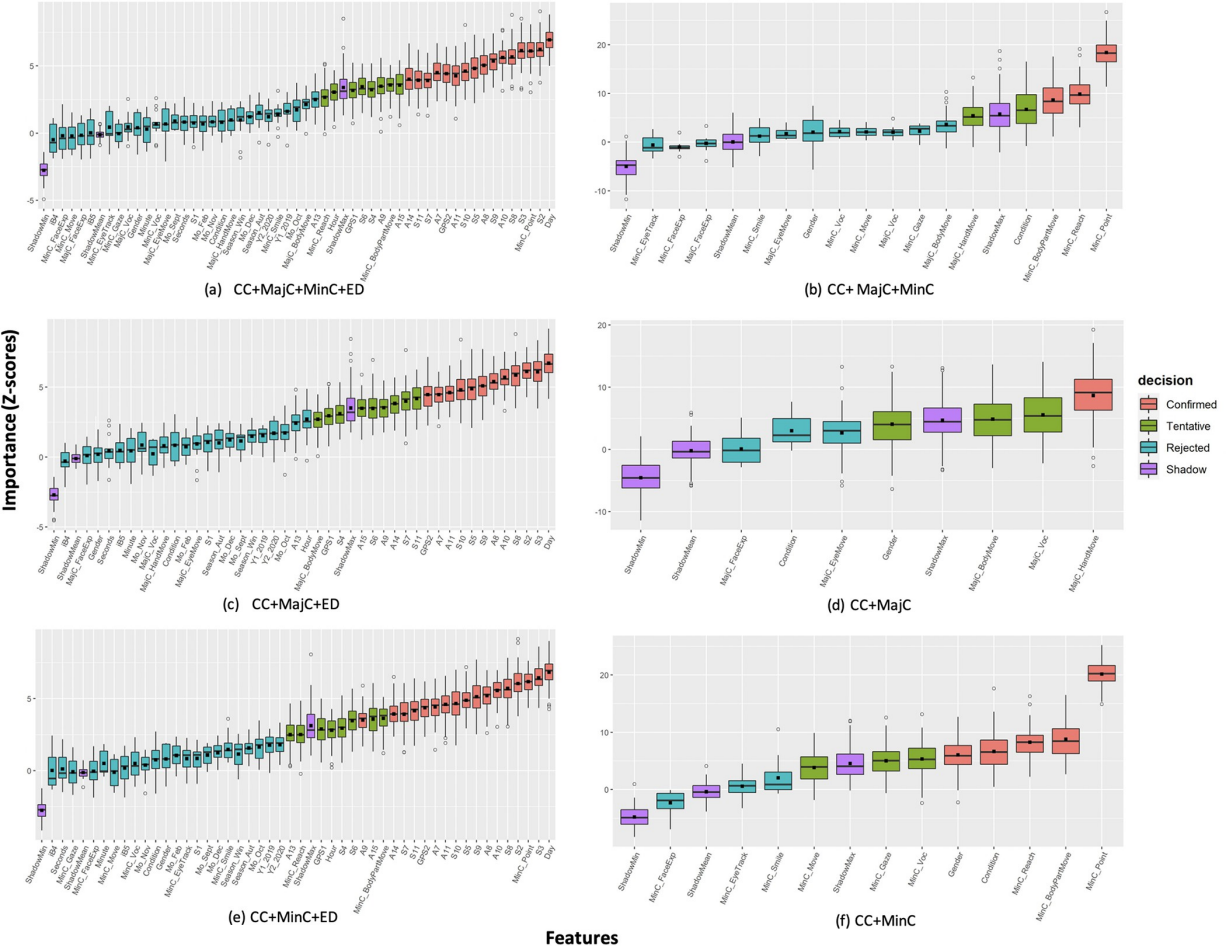
Variable importance ranking based on Boruta feature selection method in class 2. CC = child characteristics; MajC = major movement category; MinC = minor movement category; ED = environment data; Condition = PIMD or IDs; MinC_Gaze = gazing; MinC_ChangeLOS = changing line of sight; MinC_FaceExp = facial expression (other than smile); MinC_Voc = vocalization as minor category; MinC_Point = pointing; MinC_Reach = reaching; MinC_Move = moving; MinC_Appro = approaching; MinC_BodyPartMove = movement of a part of the body; MajC_EyeMove = eye movement; MajC_FaceExp = facial expressions; MajC_Voc = vocalization as major category; MajC_HandMove = hand movements; MajC_BodyMove = body movements; GPS1: Longitude; GPS2: Latitude; iB4 = classroom; iB5 = other iBeacon device; S1: Ultraviolet (UV) range (mW/cm2); S2, S3, S4: 6-axis (Accel+Geomag) sensor ranges [g]; S5, S6, S7: 6-axis (Accel+Geomag) sensor resolutions [μT]; S8: UV resolution [Lx]; S9: Pressure sensor range (hPa); S10: Temperature and humidity sensor range (°C); S11: Temperature and humidity sensor resolution (%RH); A7: Minimum temperature (°C); A8: Maximum temperature (°C); A9: Atmospheric pressure (hPa); A10: Main temperature (°C); A11: Humidity (%); A13: Cloudiness (%); A14: Wind direction (degrees); A15: Wind speed (meters/second); Mo_Feb = February; Mo_Sept = September; Mo_Oct = October; Mo_Nov = November; Mo_Dec = December; ShadowMin = minimum Z-score of a shadow attribute; ShadowMax = minimum Z-score of a shadow attribute; ShadowMean = average Z-score of a shadow attribute.

**Fig 7:**
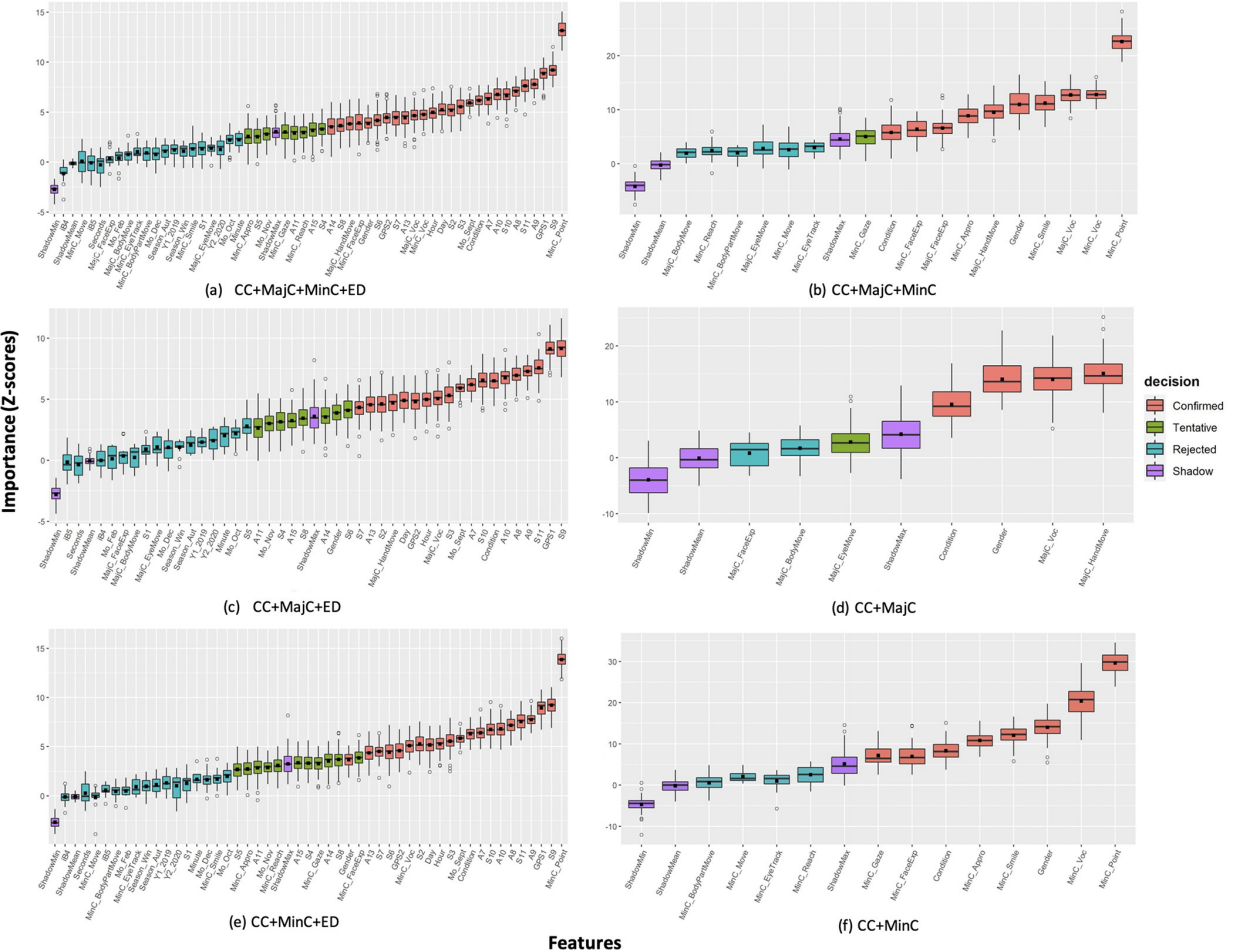
Variable importance ranking based on Boruta feature selection method in class 3. CC = child characteristics; MajC = major movement category; MinC = minor movement category; ED = environment data; Condition = PIMD or IDs; MinC_Gaze = gazing; MinC_ChangeLOS = changing line of sight; MinC_FaceExp = facial expression (other than smile); MinC_Voc = vocalization as minor category; MinC_Point = pointing; MinC_Reach = reaching; MinC_Move = moving; MinC_Appro = approaching; MinC_BodyPartMove = movement of a part of the body; MajC_EyeMove = eye movement; MajC_FaceExp = facial expressions; MajC_Voc = vocalization as major category; MajC_HandMove = hand movements; MajC_BodyMove = body movements; GPS1: Longitude; GPS2: Latitude; iB4 = classroom; iB5 = other iBeacon device; S1: Ultraviolet (UV) range (mW/cm2); S2, S3, S4: 6-axis (Accel+Geomag) sensor ranges [g]; S5, S6, S7: 6-axis (Accel+Geomag) sensor resolutions [μT]; S8: UV resolution [Lx]; S9: Pressure sensor range (hPa); S10: Temperature and humidity sensor range (°C); S11: Temperature and humidity sensor resolution (%RH); A7: Minimum temperature (°C); A8: Maximum temperature (°C); A9: Atmospheric pressure (hPa); A10: Main temperature (°C); A11: Humidity (%); A13: Cloudiness (%); A14: Wind direction (degrees); A15: Wind speed (meters/second); Mo_Feb = February; Mo_Sept = September; Mo_Oct = October; Mo_Nov = November; Mo_Dec = December; ShadowMin = minimum Z-score of a shadow attribute; ShadowMax = minimum Z-score of a shadow attribute; ShadowMean = average Z-score of a shadow attribute.

**Fig 8:**
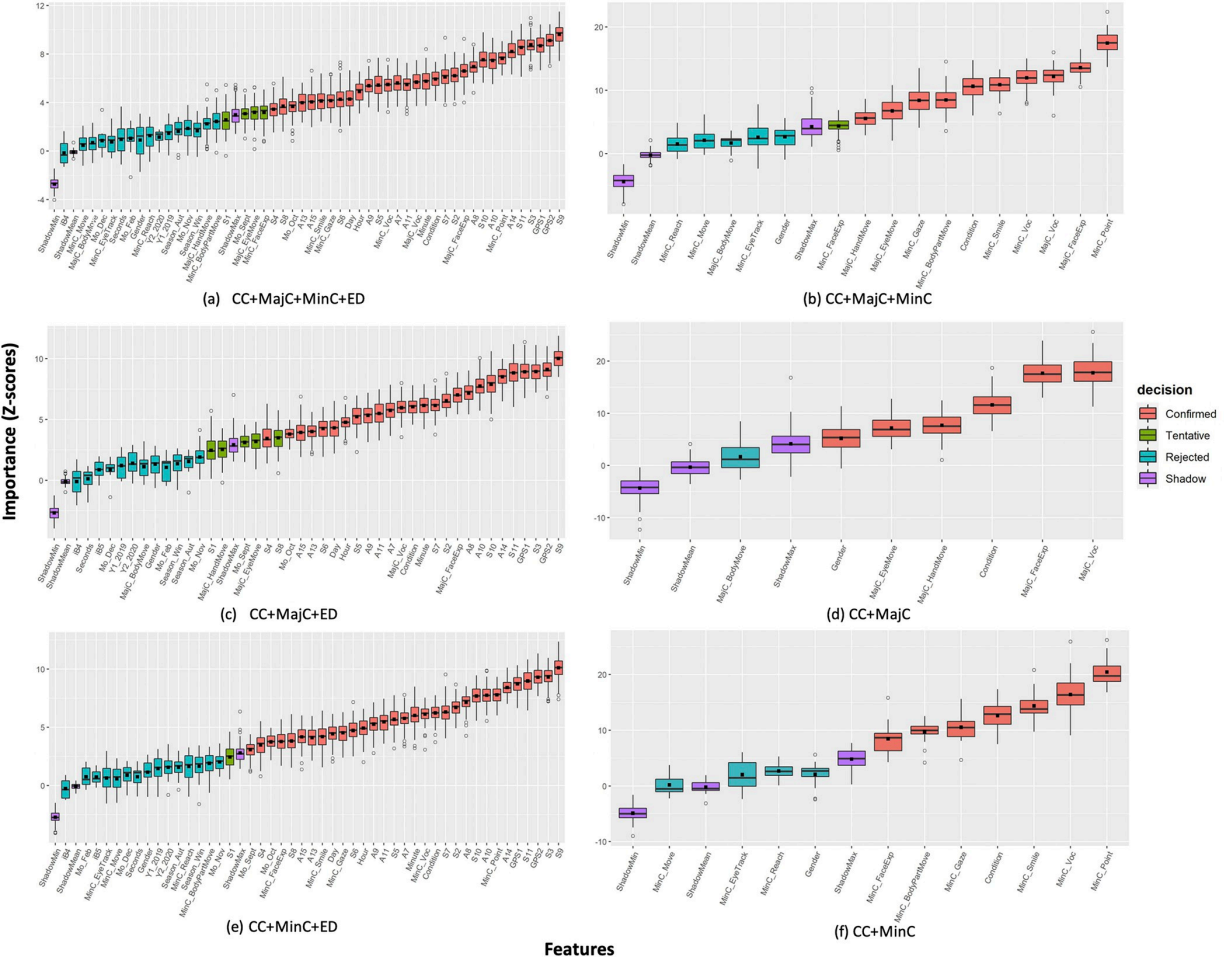
Variable importance ranking based on Boruta feature selection method in class 7. CC = child characteristics; MajC = major movement category; MinC = minor movement category; ED = environment data; Condition = PIMD or IDs; MinC_Gaze = gazing; MinC_ChangeLOS = changing line of sight; MinC_FaceExp = facial expression (other than smile); MinC_Voc = vocalization as minor category; MinC_Point = pointing; MinC_Reach = reaching; MinC_Move = moving; MinC_Appro = approaching; MinC_BodyPartMove = movement of a part of the body; MajC_EyeMove = eye movement; MajC_FaceExp = facial expressions; MajC_Voc = vocalization as major category; MajC_HandMove = hand movements; MajC_BodyMove = body movements; GPS1: Longitude; GPS2: Latitude; iB4 = classroom; iB5 = other iBeacon device; S1: Ultraviolet (UV) range (mW/cm2); S2, S3, S4: 6-axis (Accel+Geomag) sensor ranges [g]; S5, S6, S7: 6-axis (Accel+Geomag) sensor resolutions [μT]; S8: UV resolution [Lx]; S9: Pressure sensor range (hPa); S10: Temperature and humidity sensor range (°C); S11: Temperature and humidity sensor resolution (%RH); A7: Minimum temperature (°C); A8: Maximum temperature (°C); A9: Atmospheric pressure (hPa); A10: Main temperature (°C); A11: Humidity (%); A13: Cloudiness (%); A14: Wind direction (degrees); A15: Wind speed (meters/second); Mo_Feb = February; Mo_Sept = September; Mo_Oct = October; Mo_Nov = November; Mo_Dec = December; ShadowMin = minimum Z-score of a shadow attribute; ShadowMax = minimum Z-score of a shadow attribute; ShadowMean = average Z-score of a shadow attribute.

*Variable importance*. Figs 6–8 show the ranking (based on Z-scores) of randomly permuted 53 baseline features in each recalibrated datasets used to classify the movements to 2, 3, and 7 behavior outcome classes. The color-coded boxplots based on 3-level attribute weights: confirmed important (red), tentative (green), or rejected (blue), and minimum, average, and maximum shadow feature values (pink). More than half (55.6%) of the 36 ED and (9; 60%) 15 movement features, and the two child characteristics features (gender and conditions) were confirmed important features in classifying the movements to binary behavior outcome class. In recalibrated datasets with ED, timestamp-derived data, day, showed the highest importance (CC+MinC+MajC+ED, CC+MajC+ED, and CC+MinC+ED) (Fig 6A, 6C and 6E). Pointing movements (MinC_Point), GPS (latitude) location data and 10 weather indices (6-axis acceleration and geomagnetic sensor ranges [S2, S3] and resolution [S5], UV [S8], atmospheric pressure [S9], minimum [A7], maximum [A8], and main temperature [S10 and A10], and humidity [A11]) features also showed confirmed importance in all recalibrated datasets with ED. The other 3 weather indices (6-axis acceleration and geomagnetic sensor resolution [S7], humidity [S11], and wind direction [A14]) were found confirmed important when ED were added to both major and minor movement categories (CC+MajC+MinC) (Fig 5A), and minor movement categories (CC+MinC) (Fig 5E). Further, other ED features (time: hours, wind speed [A15], cloudiness [A13], atmospheric pressure [A9], other remaining acceleration and geomagnetic indices [S4, and S6], and GPS1: longitude), movements related to any parts of the body (MinC_BodyPartMove and MajC_BodyMove), and hand reaching movements (MinC_Reach) were found tentatively important features in training recalibrated movement datasets with ED.

In recalibrated datasets without ED, 6 (40%) of the 15 major and minor movement features showed importance (Fig 6B, 6D and 6F). Pointing movements (MinC_Point) of the hands and other movements related to the hands in general (MajC_HandMove), proved to be the most important features in all recalibrated movement datasets without ED (Fig 6B, 6D and 6F). Movements features related of any parts of the body (MinC_BodyPartMove) and reaching movements (MinC_Reach) in recalibrated major and minor (CC+MajC+MinC) (Fig 5B), or in minor-movements-only datasets (CC+MinC) (Fig 6F) also showed high importance. Further, child characteristics features, gender, and conditions, also showed importance in training recalibrated movement datasets without ED. Other movement features related to major (MajC_Voc) and minor (MinC_Voc) vocalizations, major body movements (MajC_BodyMove), and gaze movements (MinC_Gaze) also showed tentative importance in classifying movements to 2 behavior outcome classes.

Compared with binary classification feature selection results, Boruta performed better for 3 behavior outcome classification with almost two-thirds (n = 37; 70%) of the 53 baseline features showed significant importance (Fig 7). Hand pointing movements (MinC_Point), body movements (MajC_HandMove), and atmospheric pressure (S9) showed the highest importance in all the recalibrated datasets. In the 3 recalibrated movement datasets with ED, confirmed importance features were child characteristics features of gender (except in CC+MinC+ED) and conditions, GPS location data (longitude [GPS1] and latitude [GPS2]), 11 weather indices (6-axis acceleration and geomagnetic sensor ranges [S2, S3] and resolution [S7], atmospheric pressure [S9, A9], temperature indices [S10, A7, A8, and A10], cloudiness [S13], and 3 timestamp-derived data (day, hour, and September) (Fig 7A, 7C and 7E). The remaining 8 ED features (except iBeacon [iB4, iB5], UV [S1], time [mins and seconds], seasons, years, and months [October, and December to February] showed tentative importance in the 3 recalibrated datasets with ED. Important movement features namely vocalizations (MajC_Voc and MinC_Voc), facial (MinC_FacialExp), eye (MinC_Gaze), hand (MajC_HandMove, MinC_Point, MinC_Reach), and body movements (approaching) (MinC_Appro) showed importance in training 2 out of the 3 recalibrated datasets with ED in classifying 3 behavior outcome classes. In training major and/or minor (or both) movement recalibrated datasets without ED, gender and condition, and 8 out of 15 (53%) movement features (gazing [MinC_Gaze], smiling [MinC_Smile], facial expressions [MajC and MinC_FaceExp], eye movements [MajC_EyeMove], vocalizations [MajC and MinC_Voc], and hand [MajC_HandMove] and body movements [approach; MinC_Appro]) showed confirmed and tentative importance in classifying movements to 3 behavior outcome classes (Fig 7B, 7D and 7F).

With almost three-fourths (75%) of the 53 baseline features, Boruta feature selection algorithm performed with nearly identical results between 3 and 7 classification outcome classes. Atmospheric pressure (S9), and hand pointing movements (MinC_Point) showed the highest importance in recalibrated movement datasets with and without ED, together with vocalizations (MajC_Voc) (Fig 8). Among the baseline features, all the 20 weather index and 5 timestamp-derived data (day, hour, minutes, and the months of September and October) features showed importance in training recalibrated movement datasets with ED (Fig 8A, 8C and 8E). Further, important movement features in training either movement datasets with or without ED (Fig 8B, 8D and 8F) were gazing (MinC_Gaze), facial expressions (MajC_FaceExp, MinC_FaceExp, MinC_Smile), vocalizations (MajC and MinC_Voc), hand (MajC_HandMove) and body movements (MajC_BodyMove and MinC_BodyPartMove). In contrast with child condition feature showing confirmed importance in all recalibrated datasets, gender was only found important in training recalibrated datasets with major movement categories (CC+MajC) (Fig 8D). While UV (S1) surprisingly selected as tentatively important in training recalibrated movement datasets with ED, change of line-of-sight eye movement (MinC_ChangeLOS), iBeacon data (iB4 and iB5), and nearly half of the 13 timestamp-derived data (seconds, seasons, year, and months) were consistently selected as unimportant features in all behavior outcome classes.

#### 2.d. Classifier

The classifiers in each class were not significantly different as revealed by one-way ANOVA (Fig 5D). Table 4 shows that in class 2, SVM had the highest recall/sensitivity of 71.6%, RF had the highest precision (71.4%) while NN had the highest specificity (67.4%), F1 score (70.40%), and AUC (70.3%). In class 3, NN had the highest recall/sensitivity of 57.5% while SVM had the highest specificity of 81.47% and highest AUC of 73.7%. The highest precision and F1 score among the classifiers were obtained by RF (68.3% and 67.7%, respectively). Surprisingly, while all the classifiers had >89.9% specificity in classifying 7 behavior outcomes, RF classifier had the highest specificity of 90.1% in classifying 7, binary and 3 behavior outcome classes.

**Table 4:** Classification performance rates of the classifiers in each class.

	2-class					3-class					7-class				
	Rec.	Spec.	Prec.	F1	AUC	Rec.	Spec.	Prec.	F1	AUC	Rec.	Spec.	Prec.	F1	AUC
XGB	69.72	59.43	68.38	68.17	66.61	55.28	80.47	67.40	66.69	72.17	39.28	89.93	45.92	44.40	71.35
SVM	71.58	60.49	69.78	69.64	67.13	57.40	81.47	67.90	67.63	73.68	38.85	89.90	45.96	43.77	66.71
RF	69.06	64.48	71.42	68.91	68.33	57.52	81.33	68.30	67.70	73.50	40.16	90.12	47.68	45.50	71.68
NN	70.09	67.43	73.36	70.40	70.32	57.54	81.22	66.87	67.42	73.60	38.66	89.92	44.87	43.33	70.02

Note: XGB = eXtreme Gradient Boosting; SVM = support vector machine; RF = random forest; NN = neural network; Rec. = recall; Spec. = specificity; Prec. = precision; F1 = F1 score; AUC = area under the ROC curve.

**Fig 9:**
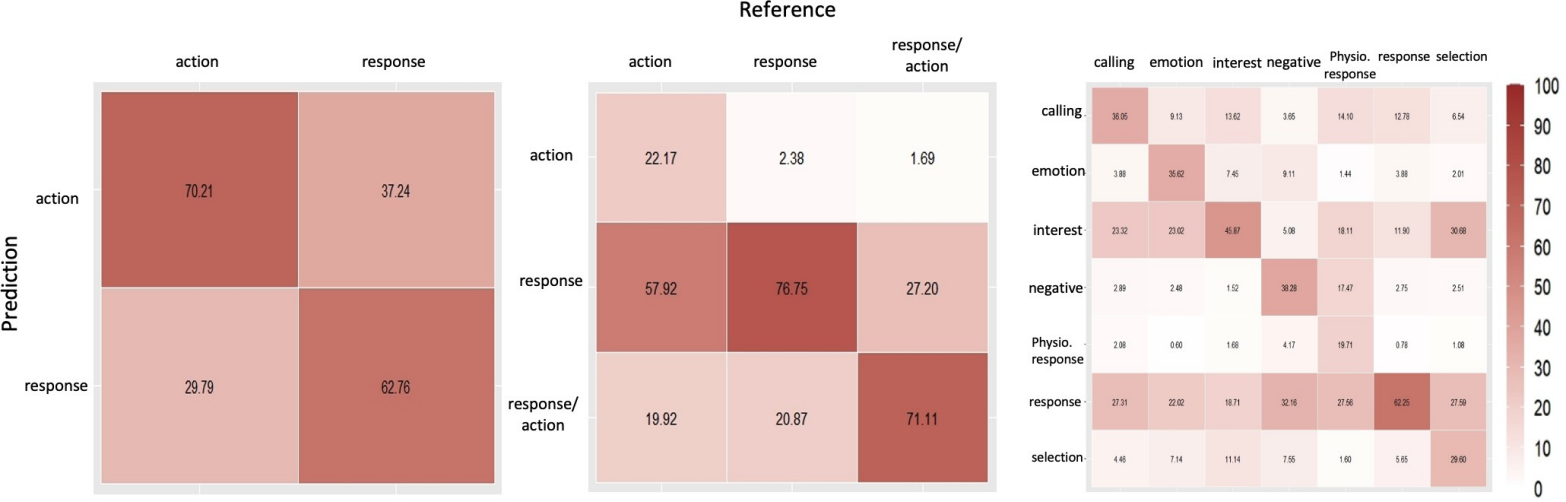
Confusion matrices showing the mean classification accuracy rates (%) in binary and multiple outcome classes.

The confusion matrix shows that the “response” and “action” behavior outcomes were classified correctly with lower confusion in binary class (Fig 9). In contrast with the “response” and “response or action” behavior outcomes, classifiers had difficulty classifying “action” behaviors which were incorrectly classified as “response” behavior outcomes. In classifying classifying 7 behavior outcomes, classifiers had incorrectly classified “physiological response” as “response”, and “selection” as “intertest” behavior outcome.

### Factor influences on the overall mean classification accuracy rate

**Fig 10:**
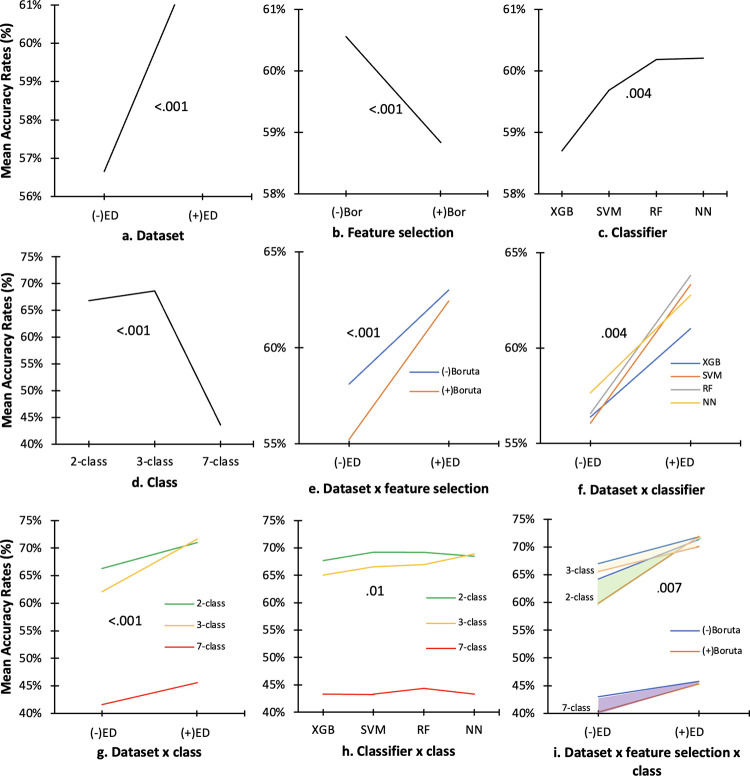
Three-way ANOVA results of the differences in the pooled mean classification accuracy rate (%) by dataset, feature selection, classifier, and class. (+) ED = dataset with environment data; (-) ED = dataset with environment data; (+) Bor = with Boruta feature selection; (-) Bor = without Boruta feature selection; XGB = eXtreme Gradient Boosting; SVM = support vector machine; RF = random forest; NN = neural network.

Three-way ANOVA revealed that recalibrated dataset, feature selection, classifier, and class had significant effects on the mean classification accuracy rates (p < 0.001) (Table 5). The high variances (η^2^) of 98% and 79% in the mean classification accuracy rates were attributed to recalibrated dataset and class, respectively. Dataset with ED (62.7%) had higher accuracy rate than dataset without ED (56.66%) (Fig 10A), non-Boruta trained (60.5%) had higher accuracy rate than Boruta-trained (58.83%), (Fig 10B), and SVM (59.7%), RF (60.2%), and NN (60.2%) classifiers had significantly higher mean classification accuracy rates than the XGB classifier (58.7%) (Fig 10C). Class 3 (68.6%) had higher accuracy rate than classes 2 (66.8%) and 7 (43.6%) (Fig 10D).

**Table 5:** Three-way ANOVA results of the influences of dataset, feature selection, classifier, and class (and interaction factors) to the mean classification accuracy rates (%).

Factors	df	F-value	η^2^
Dataset	1	351.79***	0.79
Feature Selection	1	28.29***	0.23
Classifier	3	4.77***	0.13
Class	2	2491.16***	0.98
Dataset * Feature selection	1	12.96**	0.12
Dataset * Classifier	3	4.64***	0.13
Dataset * Class	2	29.77***	0.38
Feature selection * Classifier	3	0.23	0.01
Feature selection * Class	2	0.10	0.00
Classifier * Class	6	2.88*	0.15
Dataset * Feature selection * Classifier	3	0.75	0.02
Dataset * Feature selection * Class	2	5.20**	0.10
Dataset * Classifier * Class	6	0.82	0.05
Feature selection * Classifier * Class	6	0.82	0.05
Dataset * Feature selection * Classifier * Class	6	0.81	0.05
Error	96		

Note: Dataset = recalibrated with child characteristics, major and minor behavior categories with or without environment data (ED); feature selection = with or without Boruta algorithm; classifier = XGB, SVM, RF or NN; class = class 2, 3 or 7; η^2^ = partial eta squared; adjustment for multiple comparison (Bonferroni); p < 0.001***, p < 0.01**, p < 0.05*.

Table 5 also shows that the interactions between datasets and feature selection (P < .001), between datasets and classifiers (P = 0.004), and between datasets and classes (P < .001) were found to have significant effects on the mean classification accuracy rate. Non-Boruta trained dataset with or without ED (63% and 58.1%, respectively) (Fig 10E) had higher accuracy rate than Boruta-trained dataset with or without ED (62.4% and 55.2%, respectively). Higher classification accuracy rates were also obtained by classifiers in training dataset with ED (highest using RF with accuracy of 63.8%) than training datasets without ED (highest using NN with accuracy of 62.8%) (Fig 10F). In all classes, dataset with ED had higher accuracy rates (highest in class 2: 71.6%) than dataset without ED (highest in class 3: 71%) (Fig 10G). The interaction between classifier and class also had significant effect on the the mean classification accuracy rates where the use of NN in class 2 (69%), and SVM (69%) and RF (69%) in class 3 had highest accuracy rate (Fig 10H).

The interactions among dataset, feature selection, and class (P = 0.007) also had significant effects on the mean classification accuracy rate where Boruta-trained dataset with ED in class 2 (72.13%) and non-Boruta dataset with ED in class 3 (73.49%) (Fig 10I) also had the significantly highest mean classification accuracy rates.

## Discussion

There is a significant need to support the communication of children with PIMD/IDs by interpreting their distinct and idiosyncratic movements. We proposed the development of a computer-based behavior inference AT system that interprets their movements using ML-based framework structured on utilizing different classification models, recalibrated datasets, feature selection method, and the potential use of location, weather, and time data. Herein, we take the initial step by comparing the accuracy performances of ML classifiers (XGB, SVM, RF, and NN) and the use of feature selection method (Boruta) among recalibrated dataset combinations (minor, major or both movement categories with or without environment data) on classifying the movements of children with PIMD/IDs to binary (2) or multiclass (3 and 7) behavior outcome classes using 53 features related to child movements and characteristics, and environment data (location, time and weather indices). Further, the influences of and the interactions among recalibrated dataset, feature selection, classifiers and classes on the mean classification accuracy rates were also evaluated and described.

Our study demonstrated that the recalibrated datasets in Boruta-trained minor movement dataset with environment data using the NN classifier in classifying movements to 2 behavior outcome classes and non-Boruta- trained minor movement dataset with environment data using the SVM classifier had significantly highest classification accuracy rates. Then child characteristics with either major, minor or both behavior categories) with environment data also obtained the significantly highest classification accuracy rates in classifying binary and 3 behavior outcome classes and among 16 recalibrated movement dataset combinations. Further, in classifying binary behavior outcomes, the highest mean classification accuracy rates were obtained by the Boruta-trained dataset with environment data using NN classifier and non-Boruta-trained dataset with ED using SVM and RF. When pooled, statistically significant differences in the mean classification accuracy rates were found in terms of class, the inclusion of environment data (recalibrated datasets with environment data had higher mean classification accuracy rates than those without environment data in all classes), and training datasets with Boruta feature selection (in class 7). Feature selection results revealed that among the behavior outcome classes, with almost three-fourths (70%) of the 53 baseline features, Boruta algorithm for feature selection performed better in 3 and 7 classification outcome classes with nearly identical results. Child characteristics (gender and condition), movements, location, and weather indices features were consistently selected as important features with atmospheric pressure, hand movements, vocalizations, and timestamp-derived data, day, showing the highest importance. In terms of classifiers, mean classification accuracy rates were not significantly different in all classes. Recalibrated dataset (inclusion of environment data), feature selection, classifiers, and classes had significant effects on the overall mean classification accuracy rates which indicate that higher mean classification accuracy rates were found in environment data, in non-Boruta trained dataset, in class 3, and SVM, RF, and NN classifiers. The significant interaction between datasets and feature selection, classifiers, and classes revealed that 1) non-Boruta training, 2) in all classifiers and, 3) in all classes, datasets with environment data had higher accuracy rates than dataset without environment data. The interaction between classifier and class also had significant effects on the mean classification accuracy rates where the use of NN in class 2, and SVM and RF in class 3 had the highest accuracy rates. Lastly, the interactions among recalibrated dataset, feature selection, and classes revealed that Boruta-trained dataset with environment data in class 2 and non-Boruta dataset with environment data in class 3 provided high mean classification accuracy rates.

Recalibration of the dataset has been introduced previously and found that classification accuracy could be increased when using data from the beginning of the session were used to recalibrate the model [25]. Although recalibration of the dataset was not done by dividing each session into data blocks for cross-validation of the classification and tested different decoding schemes, in our study, results show that combining child characteristics and movement categories among recalibrated dataset combinations and the pooled mean classification accuracy rates in all classes with environment data have improved the classification accuracy rates. Overall, it is also evident that the dataset with environment data had significant effects on the overall mean classification accuracy rates which indicate higher mean classification accuracy rates in the dataset with environment data. Moreover, the feature selection by Boruta revealed that environment data (location, time, and weather indices) are important features in classifying movements to 3 and 7 behavior outcome classes. Interestingly, these compare favorably with the results of a study that proposed a system for stress recognition by comparing classification performances of the recalibrated dataset by including location (proximity interactions) and weather variables (such as daily mean temperature, total precipitation, humidity, visibility, and wind speed). When each dataset was entered independently in the model (e.g. weather only, personality data only, etc.), the accuracy rates only ranged from 36.2% to 48.6%. However, when combined, the accuracy went up to 49.60%. Moreover, compared with these pairwise combinations neither performed better than their final model that combined all the datasets with location and weather data with an accuracy rate of 72.3% [45]. The performance difference in the inclusion of weather and location data in datasets for ML-based classification is further revealed in another study where models that used weather variables (e.g. precipitation intensity, precipitation probability, apparent temperature, humidity, wind speed, wind bearing, visibility, and air pressure) and location-based data obtained an average accuracy of 72.77% area under the curve (AUC) in predicting users’ activity, venue, and transportation mode [43].

Further, the interaction of the dataset with environment data with feature selection, classifiers, and classes were also found to have a significant effect on the overall mean classification accuracy rates. These suggest that regardless of training the datasets with feature selection or not, and any classifier in any class, the inclusion of environment data provides a better classification accuracy rate. The results on feature selection using Boruta algorithm proved that nearly 70% of the 53 environment data features (location, weather indices, and some timestamp-derived data) showed great importance in classifying movements to different behavior outcome classes. While atmospheric pressure was found the most important weather index feature in the three behavior outcome classes, temperature indices, humidity, acceleration and geomagnetic resolutions and ranges, wind direction, cloudiness, GPS (longitude and latitude coordinates), and timestamps-derived data (days, hours, minutes, and months) also showed confirmed importance. Noteworthy, iBeacon data (iB4 and iB5), and nearly half of the 13 timestamp-derived data (seconds, seasons, year, and months) were consistently selected as unimportant features in all behavior outcome classes. While these may provide evidence on possible association and the significant effects between location or weather indices and the movement and behavior of children with PIMD, Boruta algorithm feature selection method randomly permuted the baseline features and only identified feature importance based on attribute weights and not on index levels (high or low). Our study still needs to justify our approach and investigation on the use of these variables in interpreting the movements of children with PIMD/ID. If we know, for instance, humidity, like what has been reported in previous studies as a good predictor of children’s behavior and affective states in any season [54] also affects or is associated with the emotions of children with PIMD, an argument could be made that this could be used by our system to identify accurately the emotions, feelings or behaviors manifested when there are relatively high or low humidity levels. However, this type of argumentation and justification is lacking in this present study as it stands, thus will be addressed in our future investigation.

Our hypothesis that training the datasets with feature selection using the Boruta would improve classification accuracy performance is supported by the initial results that Boruta-trained CC+MinC+ED dataset using the NN model in class 2 (76.33%) had the highest classification accuracy rates among the recalibrated datasets and the interactions among dataset, feature selection, and class also had significant effects on the mean classification accuracy rate found Boruta-trained dataset with ED in class 2 (72.13%) had higher classification performance rates. A similar study that investigated training data with Boruta feature selection to improve classification performance found that the dataset trained with feature selection was higher (82%) than that without feature selection (74%) [27]. Although a parallel result of higher specificity and sensitivity was observed in the model with feature selection, the two classifiers were less different based on the overall accuracy [27]. However, our study also revealed contrasting results in terms of the significant interaction between feature selection, dataset, and class that the non-Boruta dataset with environment data in class 3 also had significantly higher mean classification accuracy rates. These could provide evidence that the performance of classifiers trained with feature selection using Boruta was not affected by the dataset combination as both had environment data. However, it might be sensitive to the number of classification outcomes and that other feature selection methods could best fit in training. Although our study did not compare Boruta with other feature selection methods, there have been findings that the results of a study by Chen et al (2020) could explain in part the higher accuracy rates of non-Boruta trained datasets than that of Boruta-trained ones in classifying multiclass outcomes [55]. When the results of the dataset with and without important features selection by RF methods varImp(), Boruta, and RFE were compared to get the best accuracy, Boruta was not the best feature selection method. The performance evaluation in all their experiments with three different dataset methods identified varImp()RF as the best classifier [55]. These suggest that the classifier perform better with Boruta feature selection in classifying binary outcome classes and classifiers may perform better with varImp()RF feature selection method in classifying multiclass classification tasks.

The datasets trained using NN, RF, and SVM classifiers had significantly higher mean classification accuracy rates than the dataset analyzed using XGB classifier based on its significant effect on the overall mean classification accuracy rate. This indicates that XGBoost may not be suitable for the classification task at hand despite its proven performance as an ML problem solver compared with RF for instance. A study that investigated its parameter tuning process and compared its performance by training speed and accuracy with gradient boosting and RF found that XGB and gradient boosting performed the least compared with RF [56]. The difference in the performances of XGB and RF was attributable to the use of the default parameter. While XGB and gradient boosting did not perform well using the default parameter thus require parameter search to create accurate models based on gradient boosting, RF, on the other hand, performs better when the default parameter values were used. Further, in terms of randomization, setting the subsampling rate and changing the number of features to sqrt selected at each split to reduce the size of the parameter grid to 16-fold and improving the classifier’s average performance was not necessary [56].

Similar to our study, Jha et al. (2019) also explored and compared the performances of several classifications including but not limited to SVM and RF in improving the result of binary class and multi-class problems [57]. In binary classification, the RF classifier performs better than other classifiers with a 99.29% accuracy rate [57]. However, when it comes to multiclass classification, neither SVM nor RF had the highest accuracy rates. Interestingly, our study presents contrasting results on the classification performances of the classifiers in terms of the number of classification outcomes. The interaction between classifiers and class had a significant effect on the overall accuracy rates where the use of NN in class 2 (69%), and SVM (69%) and RF (69%) in class 3 provide high accuracy rates may provide evidence that classifiers perform differently in terms of the number of classification outcomes. Both our results and of a parallel study provide similar evidence on the significant interaction between classifiers and class in terms of 3 classification problems. In the study of Bogomolov et al (2013), RF obtained a higher accuracy rate of 70.93% than NN (67.08%) in classifying a 3-class problem [58]. Interestingly, this study also provides evidence that the differences in the performances of the classifiers were also dependent on its interaction with the inclusion of environment data such as location and weather variables. The significant interaction between classifiers and dataset where higher classification accuracy rates were obtained by classifiers in training dataset with environment data than without environment data. Notably, Bogomolov et al (2013) found out that RF performed better than SVM and NN in a multiclass classification problem by combining proximity, and weather data (e.g. mean temperature, pressure, total precipitation, humidity, visibility and wind speed metrics) with personality traits and mobile phone (calls and SMS) data [58]. It is important to highlight the fact that combining weather variables and personality trait data in a model based on RF provide better accuracy (80.81%) rather using them as a single independent group for a 3-classification problem [58].

When compared by class, although NN (60.20%) had higher classification performance than RF (60.18%) and SVM (59.68%), mean comparison analyses revealed that these were not significantly different. This partially supports our hypothesis in terms of SVM-based classification with its high recall/sensitivity of 71.58% in class 2 and highest specificity of 81.47% and highest AUC of 73.68% in class 3. However, RF, as we have hypothesized, had the better performance as it not only had the highest precision (71.42%) in class 2, but it also obtained the highest precision (68.30%) and F1 score (67.70%) in class 3 and the highest performance metrics in class 7 and the highest specificity of 90.12% in classifying 7 behavior outcomes compared to binary and 3 behavior outcome classes. Several studies that compared the performance of RF with other classifiers that were also used in this study such as NN and SVM found similar results. A regression study also compared supervised ML methods like RF, SVM, and NN, in terms of a lower margin of error using mean absolute error and root mean square based on test-set found that RF has the lowest error as revealed by the highest R-square value of 96% compared with other classifiers which obtained an R-square of 93% [59]. This could be since RF, compared with other classifiers that perform better in bigger datasets, can identify patterns and consequently producing minimal errors especially in handling small datasets like ours. Compared to SVM, RF as a decision tree classification tool is efficient in handling both nominal and continuous attributes and insensitivity to data distribution assumptions with 93.36%, 93.3%, and 98.57% accuracy rates with 4, 6, and 561 features, respectively [59]. On the other hand, it can be noticed that RF’s performance increases as the number of features increases because it relies on multiple decision trees, unlike SVM which builds a model on hyperplanes based on support vectors [59]. SVM had a high accuracy rate of 96% with only 5 features than RF that had an accuracy of 95% but utilizing 9 features [19]. Further, in our study, although NN had higher overall classification performance (60.20%) than RF, the differences were not significant which are in accordance with the findings of a similar study that compared RF with NN, although accuracy rates were not significantly different, NN, on average, obtained higher accuracy rates than RF [21].

Significantly higher overall classification accuracy rates were obtained in classifying 3-class behavior outcomes (“response”, “action”, “response or action”) (68.6%) compared with classifying binary (2-class) (“response” or “action”) (66.8%) and 7-class (“calling”, “response”, “emotions”, “interest”, “negative”, “selecting”, “physiological response”) (43.6%) behavior outcomes. A decreasing trend in the results on the accuracy rates in the multiclass classification (3 and 7) was also observed in a study of several functional reaching movements from the same limb to create a versatile system for rehabilitation which also found decreasing accuracy rates (67%, 62.7%, and 50.3%) in decoding three, four and six task outcomes, respectively [25]. Our results also show that the classifiers were able to classify “response” and “action” behavior outcome classes correctly with lesser confusion in the latter. Conversely, in the 3-behavior outcome class, classifiers these “action” behaviors were incorrectly classified as “responses”. This difference may be attributed to the definition and consequently, on the categorization of individual behavior data to the correct or appropriate outcome class. Although the definition of “action” behaviors as movement, gesture, facial expression, or vocalization that affects or influences the attention or caregiver and causes a response through action was retained and used in categorizing the behavior data in the 3- behavior outcomes classification, under certain assumptions, it can be construed that the behaviors that were categorized as “action” in the binary classification should have been classified as “response or action” in the 3-outcome class. It is difficult to explain such results, this does seem to explain the higher confusion in classifying these “actions” as “responses”. Further, classifiers had consistent less confusion in classifying “responses” in binary and 3-behavior outcome classes which seem to depend on its distinct contextual definition compared to “action”. There was also less confusion in classifying “response” class in the 7-behavior outcome class, however, it is important to note that other classes like “calling”, “negative responses”, “selecting”, and “physiological responses” were incorrectly classified as “responses”, which may suggest a need for a clearer and distinct definition or label to differentiate it from other classes with similar manifestations.

The feasibility of the models presented in this study should be further investigated and experiments must be performed to address several limitations. First, the results are limited on the behavior of children with PIMD/IDs in a school setting attending one single special school. Investigating these children in other settings and including those who are attending regular schools or healthcare facilities should be considered by future investigations. Another limitation is related to testing the hypothesis that feature selection would increase the accuracy rates of the classifiers and the reliance on training the dataset using the Boruta algorithm only. As mentioned in the discussion, future studies should compare the accuracy rates using other feature selection methods like RF methods varImp() and RFE. Further, this study was limited to the use of RF, SVM, NN, and XGB in classifying binary and multiclass behavior classification outcomes. The performances of other classifiers might yield different results in classifying the behavior of children with PIMD/IDs.

While we consider the performances to be acceptable in the initial investigation and attempt to classify the behaviors of children with PIMD/IDs, part of our future work plan is to train the NN, SVM and RF classifiers to recognize a set of behavior data with environment data in binary and 3-classes at an individual level using a viable solution for body movement tracking, Microsoft Kinect, a 3D multi-sensor camera (color, infrared projector, depth and microphone array) for motion capture and gesture recognition, as a recently developed technology to capture human movements [60] and motion analysis software (Kinovea). Based on the premise of image processing and movement recognition that will be incorporated in our inference system, we will investigate utilizing discrete orthogonal polynomials which are the most used in image processing due to its ability to robustly extract distinct features from signals efficiently with low processing times which are quantified energy compaction, controlling redundancy, numerical stability, and localization [61–63]. Since several types of discrete orthogonal polynomials like Charlie polynomials, Krawtchouk polynomials, and recursive algorithm of Meixner polynomials facing challenges caused by numerical instability of coefficients for high-order polynomials, very recent studies have proposed new recurrence algorithms [61–63]. When compared with existing recurrence algorithms, the proposed algorithms exhibit better performance in generating polynomials (ranging from 8.5 to 50 times faster) with an improvement ratio of the computed coefficients ranging from 18.64 and 1269% generated sizes, which proved effective tool in image processing analyses in our behavior inference system. The output of which will be transferred to a cloud database which will consequently transmit information to the Friendly voice output communication aid (VOCA) that will produce the specific sound or voice [39]. Our last goal is to allow smart speakers to respond to the children’s behaviors, needs or desires either by sending voice commands produced by Friendly VOCA to home gadgets or appliances (eg, entertainment, lighting, or air conditioning systems) or to inform the caregivers of the need for support and assistance.

## Conclusions

Our study demonstrated the feasibility of classifying the movements of children with PIMD/IDs into binary and multiclass behavior outcomes. Analyses of variances indicated that relatively higher accuracy rates can be obtained adding environment data to any recalibrated dataset (child characteristics with major, minor (or both) movement categories). Moreover, feature selection method performed using Boruta algorithm also provided evidence that environment data (location, weather indices, and timestamp-derived data), child characteristics and movements are important features in classifying movements to different behavior outcome classes. We also found out that the improvement in the overall classification accuracy rates is also dependent on the interaction between the classifiers and classes, and the interactions among dataset, feature selection, and classes. Most importantly, the use of NN classifier in class 2, SVM and RF in class 3, and Boruta-trained dataset with environment data in classifying 2 behavior outcomes, and non-Boruta dataset with environment data in class 3 likely enabled significantly increased (>73.3%) overall mean classification accuracy rates. This highlights that although our results may be promising, classification accuracy performance could still be significantly improved to achieve optimal performance for the system that we are developing. Most importantly, this study provides the evidence that classifying the movements of children with neurological and/or motor and physical impairments can be potentially support not only for disorder-related evaluation and/or pre-clinical screening and triage, diagnosis, and treatment and intervention but also for communication.

## Supporting information

S1 File Classification accuracy rates of all the algorithms in each dataset combination statistical analysis. (PDF)

S2 File Results of the classification accuracy rates of the classifiers in each recalibrated dataset combination. (PDF)
