# Supplementary material for: Machine learning-based classification of the movements of children with profound or severe intellectual or multiple disabilities using environment data features
Source: PLoS One. 2022 Jun 30;17(6):e0269472. doi: 10.1371/journal.pone.0269472 (PMC9246124; doi:10.1371/journal.pone.0269472)
Supplement: S1 File — (PDF) [file pone.0269472.s001.pdf]

S1 File. Classification accuracy rates of the classifiers in each recalibrated dataset combination statistical analysis.

Statistical analysis

To identify the dataset combination with the highest accuracy rate (%), we conducted a multi-stage comparison using one-way ANOVA (2-tailed) with Bonferroni posthoc test for multicategory variables (classifier: XGB, SVM, RF, NN; 6 recalibrated dataset combinations within class (18 in total): CC+MajC+ED, etc.) while for binary variables (feature selection: Boruta-trained vs non-Boruta-trained; recalibrated dataset: child characteristics with minor, major, or both behavior categories, with or without ED) we used independent t-test.

In each recalibrated dataset combination, the mean accuracy rates of:

1. classifiers were compared within Boruta-trained (+ Boruta) or Non-Boruta-trained (- Boruta)
2. each classifier with Boruta was compared with the same classifier without Boruta (FS) (e.g. XGB+Boruta vs XGB-Boruta in CC+MajC+ED);
3. of each recalibrated dataset combination (+Boruta combined with -Boruta) with environment data (+ED) and without environment data (-ED) (e.g. CC+MajC+ED vs CC+MajC) per classifier.
