# Supplementary material for: Machine learning-based classification of the movements of children with profound or severe intellectual or multiple disabilities using environment data features
Source: PLoS One. 2022 Jun 30;17(6):e0269472. doi: 10.1371/journal.pone.0269472 (PMC9246124; doi:10.1371/journal.pone.0269472)
Supplement: S2 File — (PDF) [file pone.0269472.s002.pdf]

**S2 File.** Results of the classification accuracy rates of the classifiers in each recalibrated dataset combination

**Results**

1. One-way ANOVA results found no significant differences in the mean classification accuracy rates among the classifiers within Boruta-trained (+ Boruta) or Non-Boruta-trained (- Boruta).
2. There were also no significant differences in the mean classification accuracy rates of each and in each classifier with or without Boruta in each recalibrated dataset combination as revealed by independent t -test mean comparison analyses.
3. The mean classification accuracy rates (M ranged from 68% to 71%) of all Boruta-based classifiers with environment data (CC+MajC+ED) were significantly higher (P<.05) than that of all Boruta-based algorithms without ED (M range: 56% to 62%) in class 2 (Table S2.1). Further, the mean classification accuracy rate of non-Boruta based RF (72%) with ED (CC+MajC+ED) was significantly (P = .01) higher than the mean classification accuracy rate of non-Boruta RF without ED (59%) in class 2 (Table S2.1). Still in class 2, the mean classification accuracy rates (M ranged from 70% to 76%) of all the Boruta-based algorithms with ED (CC+MinC+ED) were significantly (P<.05) higher than that of non-Boruta trained dataset without ED (M range: 59% to 62%) (Table S2.2). Among the classifiers, only non-Boruta SVM with ED (CC+MinC) had significantly higher (P=.03) mean classification accuracy rate (72%) than that of non-Boruta SVM without ED in class 2 (Table S2.2). In the same class, all algorithms except Boruta-based XGB with ED (CC+MajC+MinC+ED) had significantly (P<.05) higher mean classification accuracy rates (M range: 69% to 75%) than Boruta-based dataset without ED (M range: 61% to 62%) (Table S2.3). Significant differences (P= 0.04) were also found in Boruta-trained dataset SVM with ED (CC+MajC+MinC+ED) (M = 0.49) and Boruta-based SVM without ED (39%) in class 7 (Table S2.9).

**Table S2.1.** Classification accuracy rates of the classifiers in each recalibrated dataset combination in class 2.

| | CC+MajC+ED | | | | | CC+MajC | | | | | | |
|---|---|---|---|---|---|---|---|---|---|---|---|---|
| | (+) Boruta | | (-) Boruta | | Diff. | (+) Boruta | | (-) Boruta | | Diff. (P-value) | | |
| | Acc. | P | Acc. | P | FS | Acc. | P | Acc. | P | FS | (+)ED | (-)ED |
| XGB | 67.61 | 0.79 | 66.93 | 0.62 | 0.89 | 56.25 | 0.53 | 61.86 | 0.65 | 0.18 | 0.03 | 0.26 |
| SVM | 69.47 | | 72.27 | | 0.62 | 56.40 | | 62.16 | | 0.14 | 0.01 | 0.26 |
| RF | 70.87 | | 72.16 | | 0.76 | 58.11 | | 59.09 | | 0.86 | 0.01 | 0.01 |
| NN | 72.50 | | 71.33 | | 0.79 | 62.01 | | 64.47 | | 0.55 | 0.04 | 0.08 |

Note: CC = child characteristics; MajC = major behavior category; MinC = minor behavior category; ED = environment data; Acc = accuracy; P = p-value; (+) Boruta = with Boruta feature selection; (-)Boruta = without Boruta feature selection; Diff. = difference; XGB = eXtreme Gradient Boosting; SVM = support vector machine; RF = random forest; NN = neural network.

**Table S2.2.** Classification accuracy rates of the classifiers in each recalibrated dataset combination in class 2.

| | CC+MinC+ED | | | | | CC+MinC | | | | | | |
|---|---|---|---|---|---|---|---|---|---|---|---|---|
| | (+) Boruta | | (-) Boruta | | Diff. | (+) Boruta | | (-) Boruta | | Diff. (P-value) | | |
| | Acc. | P | Acc. | P | FS | Acc. | P | Acc. | P | FS | (+)ED | (-)ED |
| XGB | 70.42 | 0.65 | 67.73 | 0.60 | 0.64 | 59.39 | 0.91 | 66.74 | 0.78 | 0.09 | 0.01 | 0.86 |
| SVM | 74.05 | | 72.08 | | 0.69 | 60.19 | | 62.80 | | 0.57 | 0.02 | 0.03 |
| RF | 71.70 | | 74.17 | | 0.60 | 58.94 | | 65.15 | | 0.12 | 0.01 | 0.05 |
| NN | **76.33** | | 72.46 | | 0.42 | 61.55 | | 66.78 | | 0.20 | 0.00 | 0.21 |

Note: CC = child characteristics; MajC = major behavior category; MinC = minor behavior category; ED = environment data; Acc = accuracy; P = p-value; (+) Boruta = with Boruta feature selection; (-)Boruta = without Boruta feature selection; Diff. = difference; XGB = eXtreme Gradient Boosting; SVM = support vector machine; RF = random forest; NN = neural network.

**Table S2.3.** Classification accuracy rates of the classifiers in each recalibrated dataset combination in class 2.

| | CC+MajC+MinC+ED | | | | | CC+MajC+MinC | | | | | | |
|---|---|---|---|---|---|---|---|---|---|---|---|---|
| | (+) Boruta | | (-) Boruta | | Diff. | (+) Boruta | | (-) Boruta | | Diff. (P-value) | | |
| | Acc. | P | Acc. | P | FS | Acc. | P | Acc. | P | FS | (+)ED | (-)ED |
| XGB | 69.05 | 0.68 | 68.18 | 0.61 | 0.87 | 61.59 | 0.99 | 64.51 | 0.75 | 0.49 | 0.14 | 0.41 |
| SVM | 72.77 | | 71.67 | | 0.83 | 60.64 | | 63.67 | | 0.50 | 0.04 | 0.05 |
| RF | 72.31 | | 73.56 | | 0.82 | 61.59 | | 65.80 | | 0.28 | 0.03 | 0.13 |
| NN | 75.38 | | 73.86 | | 0.71 | 62.05 | | 67.69 | | 0.29 | 0.02 | 0.14 |

Note: CC = child characteristics; MajC = major behavior category; MinC = minor behavior category; ED = environment data; Acc = accuracy; P = p-value; (+) Boruta = with Boruta feature selection; (-)Boruta = without Boruta feature selection; Diff. = difference; XGB = eXtreme Gradient Boosting; SVM = support vector machine; RF = random forest; NN = neural network.

**Table S2.4.** Classification accuracy rates of the classifiers in each recalibrated dataset combination in class 3.

| | CC+MajC+ED | | | | | CC+MajC | | | | | | |
|---|---|---|---|---|---|---|---|---|---|---|---|---|
| | (+) Boruta | | (-) Boruta | | Diff. | (+) Boruta | | (-) Boruta | | Diff. (P-value) | | |
| | Acc. | P | Acc. | P | FS | Acc. | P | Acc. | P | FS | (+)ED | (-)ED |
| XGB | 68.01 | 0.99 | 67.56 | 0.79 | 0.93 | 62.27 | 1.00 | 64.49 | 0.97 | 0.55 | 0.15 | 0.52 |
| SVM | 68.76 | | 68.79 | | 1.00 | 62.73 | | 63.08 | | 0.92 | 0.15 | 0.18 |
| RF | 68.49 | | 71.97 | | 0.50 | 61.89 | | 63.69 | | 0.69 | 0.18 | 0.09 |
| NN | 69.36 | | 68.22 | | 0.77 | 61.89 | | 64.91 | | 0.46 | 0.09 | 0.39 |

Note: CC = child characteristics; MajC = major behavior category; MinC = minor behavior category; ED = environment data; Acc = accuracy; P = p-value; (+) Boruta = with Boruta feature selection; (-)Boruta = without Boruta feature selection; Diff. = difference; XGB = eXtreme Gradient Boosting; SVM = support vector machine; RF = random forest; NN = neural network.

**Table S2.5.** Classification accuracy rates of the classifiers in each recalibrated dataset combination in class 3.

| | CC+MinC+ED | | | | | CC+MinC | | | | | | |
|---|---|---|---|---|---|---|---|---|---|---|---|---|
| | (+) Boruta | | (-) Boruta | | Diff. | (+) Boruta | | (-) Boruta | | Diff. (P-value) | | |
| | Acc. | P | Acc. | P | FS | Acc. | P | Acc. | P | FS | (+)ED | (-)ED |
| XGB | 69.32 | 0.76 | 69.78 | 0.26 | 0.92 | 68.37 | 0.99 | 67.12 | 0.87 | 0.73 | 0.82 | 0.52 |
| SVM | 71.90 | | 76.29 | | 0.24 | 67.42 | | 69.18 | | 0.63 | 0.27 | 0.05 |
| RF | 72.71 | | 73.69 | | 0.82 | 67.87 | | 69.20 | | 0.66 | 0.25 | 0.18 |
| NN | 69.22 | | 72.25 | | 0.38 | 67.94 | | 69.30 | | 0.63 | 0.63 | 0.41 |

Note: CC = child characteristics; MajC = major behavior category; MinC = minor behavior category; ED = environment data; Acc = accuracy; P = p-value; (+) Boruta = with Boruta feature selection; (-)Boruta = without Boruta feature selection; Diff. = difference; XGB = eXtreme Gradient Boosting; SVM = support vector machine; RF = random forest; NN = neural network.

**Table S2.6.** Classification accuracy rates of the classifiers in each recalibrated dataset combination in class 3.

| | CC+MajC+MinC+ED | | | | | CC+MajC+MinC | | | | | | |
|---|---|---|---|---|---|---|---|---|---|---|---|---|
| | (+) Boruta | | (-) Boruta | | Diff. | (+) Boruta | | (-) Boruta | | Diff. (P-value) | | |
| | Acc. | P | Acc. | P | FS | Acc. | P | Acc. | P | FS | (+)ED | (-)ED |
| XGB | 70.51 | 0.98 | 71.48 | 0.72 | 0.83 | 66.59 | 1.00 | 67.10 | 0.92 | 0.89 | 0.35 | 0.29 |
| SVM | 71.32 | | 75.38 | | 0.22 | 66.17 | | 69.20 | | 0.45 | 0.16 | 0.11 |
| RF | 71.56 | | 74.17 | | 0.58 | 66.98 | | 67.83 | | 0.83 | 0.32 | 0.15 |
| NN | 70.15 | | 72.29 | | 0.46 | 66.61 | | 69.32 | | 0.40 | 0.23 | 0.35 |

Note: CC = child characteristics; MajC = major behavior category; MinC = minor behavior category; ED = environment data; Acc = accuracy; P = p-value; (+) Boruta = with Boruta feature selection; (-)Boruta = without Boruta feature selection; Diff. = difference; XGB = eXtreme Gradient Boosting; SVM = support vector machine; RF = random forest; NN = neural network.

**Table S2.7.** Classification accuracy rates of the classifiers in each recalibrated dataset combination in class 7.

| | CC+MajC+ED | | | | | CC+MajC | | | | | | |
|---|---|---|---|---|---|---|---|---|---|---|---|---|
| | (+) Boruta | | (-) Boruta | | Diff. | (+) Boruta | | (-) Boruta | | Diff. (P-value) | | |
| | Acc. | P | Acc. | P | FS | Acc. | P | Acc. | P | FS | (+)ED | (-)ED |
| XGB | 43.86 | 0.97 | 45.33 | 0.98 | 0.76 | 39.88 | 0.90 | 43.57 | 0.98 | 0.27 | 0.33 | 0.68 |
| SVM | 44.73 | | 44.64 | | 0.98 | 39.45 | | 44.53 | | 0.21 | 0.19 | 0.97 |
| RF | 45.20 | | 46.20 | | 0.79 | 41.44 | | 43.35 | | 0.50 | 0.26 | 0.41 |
| NN | 43.42 | | 44.69 | | 0.73 | 41.42 | | 44.33 | | 0.42 | 0.59 | 0.92 |

Note: CC = child characteristics; MajC = major behavior category; MinC = minor behavior category; ED = environment data; Acc = accuracy; P = p-value; (+) Boruta = with Boruta feature selection; (-)Boruta = without Boruta feature selection; Diff. = difference; XGB = eXtreme Gradient Boosting; SVM = support vector machine; RF = random forest; NN = neural network.

**Table S2.8.** Classification accuracy rates of the classifiers in each recalibrated dataset combination in class 7

| | CC+MinC+ED | | | | | CC+MinC | | | | | | |
|---|---|---|---|---|---|---|---|---|---|---|---|---|
| | (+) Boruta | | (-) Boruta | | Diff. | (+) Boruta | | (-) Boruta | | Diff. (P-value) | | |
| | Acc. | P | Acc. | P | FS | Acc. | P | Acc. | P | FS | (+)ED | (-)ED |
| XGB | 44.49 | 0.86 | 47.15 | 0.81 | 0.67 | 40.29 | 0.88 | 41.73 | 1.00 | 0.72 | 0.46 | 0.27 |
| SVM | 46.09 | | 44.52 | | 0.65 | 37.74 | | 40.85 | | 0.56 | 0.07 | 0.42 |
| RF | 48.01 | | 47.61 | | 0.93 | 40.72 | | 40.81 | | 0.99 | 0.10 | 0.13 |
| NN | 44.66 | | 43.87 | | 0.86 | 40.84 | | 41.33 | | 0.90 | 0.33 | 0.59 |

Note: CC = child characteristics; MajC = major behavior category; MinC = minor behavior category; ED = environment data; Acc = accuracy; P = p-value; (+) Boruta = with Boruta feature selection; (-)Boruta = without Boruta feature selection; Diff. = difference; XGB = eXtreme Gradient Boosting; SVM = support vector machine; RF = random forest; NN = neural network.

**Table S2.9.** Classification accuracy rates of the classifiers in each recalibrated dataset combination in class 7

| | **CC+MajC+MinC+ED** | | | | | **CC+MajC+MinC** | | | | | | |
|---|---|---|---|---|---|---|---|---|---|---|---|---|
| | **(+) Boruta** | | **(-) Boruta** | | **Diff.** | **(+) Boruta** | | **(-) Boruta** | | **Diff. (P-value)** | | |
| | **Acc.** | **P** | **Acc.** | **P** | **FS** | **Acc.** | **P** | **Acc.** | **P** | **FS** | **(+)ED** | **(-)ED** |
| XGB | 43.01 | 0.63 | 47.73 | 0.82 | 0.39 | 38.75 | 0.85 | 44.33 | 1.00 | 0.09 | 0.34 | 0.46 |
| SVM | 48.85 | | 46.12 | | 0.52 | 38.66 | | 43.98 | | 0.34 | 0.04 | 0.68 |
| RF | 46.55 | | 47.45 | | 0.82 | 41.49 | | 44.25 | | 0.51 | 0.26 | 0.36 |
| NN | 45.32 | | 44.23 | | 0.78 | 41.82 | | 43.64 | | 0.73 | 0.51 | 0.87 |

Note: CC = child characteristics; MajC = major behavior category; MinC = minor behavior category; ED = environment data; Acc = accuracy; P = p-value; (+) Boruta = with Boruta feature selection; (-)Boruta = without Boruta feature selection; Diff. = difference; XGB = eXtreme Gradient Boosting; SVM = support vector machine; RF = random forest; NN = neural network.
